# *sodC*-Based Real-Time PCR for Detection of *Neisseria meningitidis*

**DOI:** 10.1371/journal.pone.0019361

**Published:** 2011-05-05

**Authors:** Jennifer Dolan Thomas, Cynthia P. Hatcher, Dara A. Satterfield, M. Jordan Theodore, Michelle C. Bach, Kristin B. Linscott, Xin Zhao, Xin Wang, Raydel Mair, Susanna Schmink, Kathryn E. Arnold, David S. Stephens, Lee H. Harrison, Rosemary A. Hollick, Ana Lucia Andrade, Juliana Lamaro-Cardoso, Ana Paula S. de Lemos, Jenna Gritzfeld, Stephen Gordon, Ahmet Soysal, Mustafa Bakir, Dolly Sharma, Shabnam Jain, Sarah W. Satola, Nancy E. Messonnier, Leonard W. Mayer

**Affiliations:** 1 Meningitis and Vaccine Preventable Diseases Branch, Division of Bacterial Diseases, National Center for Immunization and Respiratory Diseases, Centers for Disease Control and Prevention, Atlanta, Georgia, United States of America; 2 Division of Public Health, Georgia Department of Community Health, Atlanta, Georgia, United States of America; 3 Emory University School of Medicine, Atlanta, Georgia, United States of America; 4 Georgia Emerging Infections Program, Atlanta, Georgia, United States of America; 5 Veterans Affairs Medical Center, Atlanta, Georgia, United States of America; 6 Children's Healthcare of Atlanta, Atlanta, Georgia, United States of America; 7 Biology Department, Agnes Scott College, Decatur, Georgia, United States of America; 8 Johns Hopkins Bloomberg School of Public Health, Baltimore, Maryland, United States of America; 9 Instituto de Patologia Tropical e Saúde Pública, Universidade Federal de Goiás, Goiânia, Goiás, Brazil; 10 Instituto Adolfo Lutz, São Paulo, Brazil; 11 Respiratory Infection, Clinical Group, Liverpool School of Tropical Medicine, Liverpool, United Kingdom; 12 Division of Pediatric Infectious Diseases, Marmara University School of Medicine, Istanbul, Turkey; Hopital Raymond Poincare - Universite Versailles St. Quentin, France

## Abstract

Real-time PCR (rt-PCR) is a widely used molecular method for detection of *Neisseria meningitidis* (Nm). Several rt-PCR assays for Nm target the capsule transport gene, *ctrA*. However, over 16% of meningococcal carriage isolates lack *ctrA*, rendering this target gene ineffective at identification of this sub-population of meningococcal isolates. The Cu-Zn superoxide dismutase gene, *sodC*, is found in Nm but not in other *Neisseria* species. To better identify Nm, regardless of capsule genotype or expression status, a *sodC*-based TaqMan rt-PCR assay was developed and validated. Standard curves revealed an average lower limit of detection of 73 genomes per reaction at cycle threshold (C_t_) value of 35, with 100% average reaction efficiency and an average R^2^ of 0.9925. 99.7% (624/626) of Nm isolates tested were *sodC*-positive, with a range of average C_t_ values from 13.0 to 29.5. The mean *sodC* C_t_ value of these Nm isolates was 17.6±2.2 (±SD). Of the 626 Nm tested, 178 were nongroupable (NG) *ctrA*-negative Nm isolates, and 98.9% (176/178) of these were detected by *sodC* rt-PCR. The assay was 100% specific, with all 244 non-Nm isolates testing negative. Of 157 clinical specimens tested, *sodC* detected 25/157 Nm or 4 additional specimens compared to *ctrA* and 24 more than culture. Among 582 carriage specimens, *sodC* detected Nm in 1 more than *ctrA* and in 4 more than culture. This *sodC* rt-PCR assay is a highly sensitive and specific method for detection of Nm, especially in carriage studies where many meningococcal isolates lack capsule genes.

## Introduction

*Neisseria meningitidis* (Nm) is the etiologic agent of epidemic bacterial meningitis and rapidly fatal sepsis throughout the world. Many clinical, reference, and research laboratories must be able to rapidly detect Nm either from patients with invasive disease or from asymptomatic carriers. Bacterial isolates or clinical specimens may be sent to the laboratory from patients with suspected meningitis, while isolates or swab eluates may be the specimens that come to the laboratory from possible carriers.

Common techniques employed for the identification of Nm include biochemical tests, slide agglutination serogrouping (SASG) [1], [2], and the polymerase chain reaction (PCR) [3]–[6]. Chromogenic biochemical tests and SASG can be subjective, sometimes complicating species identification [5], [7].

Unlike biochemical tests and SASG, PCR does not require viable bacteria and can be used to identify and characterize even nongroupable (NG) meningococci. Additionally, it is necessary to detect small numbers of Nm in clinical specimens; bacterial loads in cerebrospinal fluid (CSF) of patients range from 3×10^1^ to 4×10^9^ CFU/ml [8], [9]. TaqMan rt-PCR has been shown to detect as few as 8 meningococcal genomes per reaction [4], [5] and results are obtained within 2.5 hours.

*ctrA* may be the most frequently targeted gene to detect Nm using PCR [10]. However, the capsule locus, including *ctrA*, is subject to rearrangement [11]–[15], and 16% or more of carried meningococci have been shown to lack *ctrA* altogether [11], [12]. Invasive NG meningococci can undergo similar rearrangements of the capsule region (J. Dolan Thomas, unpublished data), although these events may be less common than in carriage isolates.

The [Cu, Zn]-cofactored superoxide dismutase gene, *sodC*, is located 1.23 Mb from the capsule locus in the 2.27-Mb Nm serogroup B strain MC58 genome [16] and encodes the virulence factor Cu, Zn Sod. Cu, Zn Sod is a periplasmic enzyme [17], making it theoretically less susceptible to antigenic variation due to selective pressure than a cell-surface exposed molecule. *sodC* is believed to have been acquired by Nm via horizontal transfer from *Haemophilus influenzae* (Hi) [18]. There are no reports of meningococci that lack *sodC*, suggesting its importance to the survival of the organism *in vivo* and a strong selective pressure for its retention. However, *sodC* is not found in other *Neisseria* spp. [17]–[19].

The objective of this study was to improve detection of meningococci, especially of carriage isolates which may be *ctrA*-negative and NG, by developing a sensitive and specific rt-PCR assay for identification of all meningococci, regardless of capsule genotype or expression status.

## Materials and Methods

### Ethics statement

Ethics approval was not obtained from the CDC Institutional Review Board (IRB) for clinical specimens reported in this study because the specimens were sent to CDC for detection of meningitis etiology as part of reference lab functions.

CDC IRB approval was not obtained for *sodC* testing of DNA extractions from Brazil and UK carriage specimens because those extractions were not tested by CDC researchers. DNA extractions of carriage specimens were not considered human specimens by the CDC, Emory University, and Children's Hospital of Atlanta (CHOA) IRBs for this study, as they do not meet the definition of a living human subject. IRB approval was obtained by the institutions who collected the biological carriage specimens from the human subjects: (1) the National Ethics Research Committee and by the Regional Ethics Committee of the Hospital Materno Infantil, Secretary of Health of Goias State, Brazil; (2) the Emory University IRB; or (3) the National Health Service Research Ethics Committee (08/H1001/52), sponsored by the Royal Liverpool and Broadgreen University Hospitals Trust.

### Bacterial strains and culture methods

Control isolates used for assay design and optimization are defined in Table S1.

626 cell lysates were used to determine the sensitivity of the *sodC* assay (Table 1 and Table S2), including lysates prepared from a temporally and geographically dispersed convenience sample of isolates from the CDC Meningitis Laboratory strain collection (received 1993–2008, n = 106) and all isolates from a US carriage study (n = 520) [20], [21] known to be Nm by SASG [1], [2], rt-PCR serogrouping [5], NH strips (bioMérieux® sa), and Cystine Trypticase Agar (CTA) sugars (Remel) [1], [2]. To further confirm identification, multilocus sequence typing (MLST) was performed on all U.S. carriage study and *ctrA*-negative NG isolates.

**Table 1:** Summary of 626 Nm isolates used to test the sensitivity of the *sodC* assay.

Source of Strains	Number of Strains	SASG	SG-PCR^1^	*ctrA*	*ctrA* C_t_ 2	*sodC*	Avg *sodC* C_t_ 3	Additional Molecular Tests Confirming ID as Nm
Kellerman et al. [21]	25	NG	not done	−	1/25 was 36.0^4^	+	16.7±1.8	MLEE^5^ and MLST^6^
Carriage Panel	56	NG	not done	+	19.7±1.8	+	16.8±2.3	MLEE and MLST
	33	B	not done	+	21.2±1.0	+	16.6±1.3	MLEE and MLST
	2	C	not done	+	21.4±0.5	+	15.0±0.5	MLEE and MLST
	75	Y	not done	+	22.1±1.2	+	17.7±1.4	MLEE and MLST
	3	Z	not done	+	19.7±1.2	+	16.7±2.1	MLEE and MLST
Clark et al. [20]	43	NG	NG	−	No C_t_	+	16.5±0.9	MLST
GA Carriage Panel	2	NG	NG	−	No C_t_	−	No C_t_	MLST
	4	NG	B	−	No C_t_	+	17.7±0.9	MLST
	1	NG	X	−	No C_t_	+	17.7	MLST
	5	NG	Y	−	No C_t_	+	17.6±0.5	MLST
	37	NG	NG	+	17.9±3.1	+	16.7±0.8	MLST
	19	NG	B	+	18.1±1.4	+	17.2±1.1	MLST
	1	NG	C	+	16.7	+	17.0	MLST
	9	NG	Y	+	17.1±1.0	+	17.0±0.9	MLST
	14	B	B	+	16.5±0.9	+	15.8±0.6	MLST
	6	Y	Y	+	17.8±1.0	+	17.7±0.8	MLST
	1	29E	n/a^8^	+	14.2	+	16.6	MLST
Clark et al. [20]	71	NG	NG	−	No C_t_	+	17.0±1.5	MLST
MD Carriage Panel	1	NG	Y	−	No C_t_	+	17.2	MLST
	29	NG	NG	+	16.5±1.2	+	16.9±1.2	MLST
	36	NG	B	+	17.6±2.6	+	17.7±1.1	MLST
	3	NG	C	+	16.9±2.0	+	17.9±0.3	MLST
	25	NG	Y	+	18.0±2.1	+	17.5±1.4	MLST
	19	Y	Y	+	18.1±2.2	+	17.6±1.6	MLST
CDC Strain	26	NG	NG	−	No C_t_	+	20.0±1.5	MLST
Collection	1	NG	Y	+	20.0	+	19.4	
	11	A	A	+	17.5±1.7	+	19.0±2.4	MLST on 1/11
	10	B	B	+	17.8±1.7	+	20.5±1.8	MLST on 4/11; 1/11 serotyped and serosubtyped
	10	C	C	+	17.6±2.1	+	19.8±2.7	MLST on 5/10; 16S on 1/10
	9	W135	W135	+	22.1±3.6	+	22.3±3.5	MLST on 1/9
	1	W135	NG	+	21.2	+	23.1	
	9	X	X	+	20.4±4.5	+	25.0±3.3	MLST on 1/9
	11	Y	Y	+	16.4±2.2	+	20.5±1.6	MLST on 10/11
	9	Z	n/a^7^	+	16.7±1.8	+	19.2±1.1	MLST on 1/9; 3/9 serosubtyped, 1 also had 16S
	9	29E	n/a^8^	+	16.8±2.0	+	19.0±1.2	16S on 1/9

1 Serogroup-specific PCR testing was performed to detect serogroups A, B, C, W135, X, and Y; isolates not positive for one of these serogroups were termed NG by PCR. Serogroup-specific PCR was not performed on carriage study isolates that were *ctrA*-negative.

2 All *ctrA* C_t_ values were generated at CDC except the MD carriage isolates, which were tested at the MD Department of Health and Mental Hygiene.

3 All *sodC* results were an average of 2 C_t_ values except M17304, which is an average of 4 C_t_s.

4 24 *ctrA*-negative, *sodC*-positive NG Nm from this carriage study yielded No C_t_ for *ctrA*. However, one such isolate, M05046, gave a negative *ctrA* C_t_ value of 36.0.

5 MLEE results for the isolates collected in the Kellerman et al. carriage study were previously reported [21].

6 Where ST is given, this isolate was previously characterized [12]. Where no ST is given, MLST data is to be published elsewhere.

7 There is no Z PCR serogrouping assay currently in use in the CDC Meningitis Laboratory. However, this isolate tested negative by rt-PCR for serogroup A, B, C, W135, X, and Y genes.

8 There is no 29E PCR serogrouping assay currently in use in the CDC Meningitis Laboratory. However, this isolate tested negative by rt-PCR for serogroup A, B, C, W135, X, and Y genes.

The specificity of the *sodC* assay for detecting only meningococci was determined using cell lysates from a total of 244 non-Nm isolates (Table 2).

**Table 2:** 244 non-Nm isolates were used to test the specificity of the *sodC* assay.

Organism^1^	*n*	*sodC* ^+^
*M. catarrhalis*	22	0
*H. aphrophilus/paraphrophilus*	2	0
*H. aphrophilus*	1	0
*H. influenzae* biogroup *aegyptius*	1	0
*H. influenzae* serotype a	1	0
*H. influenzae* serotype b	1	0
*H. influenzae* serotype c	1	0
*H. influenzae* serotype d	1	0
*H. influenzae* serotype e	2	0
*H. influenzae* serotype f	1	0
*H. influenzae nontypeable* (NTHi)	80	0
*H. parainfluenzae*	10	0
*H. haemolyticus*	2	0
*N. lactamica*	93	0
*N.* spp.	3	0
*N. polysaccharea*	1	0
*N. cinerea*	2	0
*N. subflava*	1	0
*N. sicca*	2	0
*N. gonorrhoeae*	5	0
*E. coli* K1	2	0
*C. neoformans*	1	0
*S. aureus*	1	0
*S. pneumoniae*	1	0
*L. monocytogenes*	1	0
*A. pleuropneumoniae*	1	0
*S. choleraesuis*	1	0
*S. agalactiae*	1	0
*P. aeruginosa*	1	0
*C. diptheriae*	1	0
*B. pertussis*	1	0
Total	244	0

1 92 *N. lactamica*, 4 *N. gonorrhoeae*, 19 *M. catarrhalis*, and 2 *H*. spp. from this panel were collected in a Georgia Nm carriage study [20]. 3 *N*. spp., 1 *N. polysaccharea*, 2 *M. catarrhalis*, 9 *H. parainfluenzae*, 1 *H. haemolyticus*, 75 NTHi, and 1 Hie were collected in a Georgia Hib carriage study [24].

### Clinical specimens

CSF specimens from pediatric meningitis patients were cultured as soon as possible after collection. Specimens that were culture-negative were sent to CDC on ice for detection of meningitis etiology by the Marmara University School of Medicine in Istanbul, Turkey, and came from patients who met the case definition for purulent meningitis [leukocytosis (>100 cells/mm^3^) and either elevated protein (>100 mg/dl) or decreased glucose (<40 mg/dl)]. After DNA extraction and real-time PCR testing of all specimens for *ctrA* of Nm [5], *lytA* of *S. pneumoniae* [22], and *bexA* and/or *bcs2* of Hi, the subset of specimens chosen to test the *sodC* assay (n = 120) were either (1) positive for *ctrA* (n = 12) or (2) *ctrA* ^−^ *lytA* ^−^ *bexA*/*bcs2* ^−^ (n = 108).

37 U.S. clinical specimens were referred to CDC for detection or confirmation of bacterial meningitis etiology from January to June 2009, including CSF (n = 21), whole blood (n = 6), serum (n = 6), and tissues (n = 4) (Table 3).

**Table 3:** U.S. and Turkey clinical specimens tested for *sod C* and *ctrA* and compared to culture.

Specimen	Total n	n *sodC* ^+^	% sodC^+^	95% CI	n *ctrA* ^+^	% ctrA^+^	95% CI^1^	n Nm Culture Positive^2^	% Culture Positive	95% CI
CSF	141	24	17.0	11% to 24%	20	14.2	9% to 21%	1	0.7	0% to 4%
Blood	6	1	16.7	0% to 64%	1	16.7	0% to 64%	0	0.0	0% to 46%
Serum	6	0	0.0	0% to 46%	0	0.0	0% to 46%	0	0.0	0% to 46%
Body Tissue	4	0	0.0	0% to 60%	0	0.0	0% to 60%	0	0.0	0% to 60%
Total	157	25	15.9	n/a^3^	21	13.4	n/a	1	0.6	n/a

1 Exact binomial 95% confidence interval.

2 Culture was attempted for all 120 Turkey CSF specimens. With the exception of the one *ctrA*-negative, *sodC*-positive U.S. CSF specimen that was culture-positive, culture was either not attempted, not reported or, in one case, the isolate was nonviable two times for the other 36 U.S. clinical specimens.

3 n/a, not applicable.

### Carriage specimens

The carriage specimens were obtained from three carriage studies. 1. In Goiania, Brazil, nasopharyngeal (NP) swabs (n = 223) were obtained from 154 children (ranging from 2–163 months of age) attending two daycare centers, 59 adult contacts of the attendees, and 10 daycare workers. The specimens were placed into skim milk-tryptone-glucose-glycerine (STGG) transport medium [23] and sent immediately to the Applied Microbiology Laboratory of Federal University of Goiás in Brazil for processing. The vials were then kept frozen during transport to CDC, where DNA extractions and rt-PCR were performed.

2. 291 posterior NP swab specimens were collected from a random sample of children 6 to 59 months of age who presented to the Emergency Department at CHOA at Egleston from March to August, 2009 [24]. Each swab specimen was immediately placed into 1 ml STGG [23]. Specimens were transported at room temperature to the clinical microbiology laboratory within 12 hours of collection for storage at −80°C until processing. An aliquot of the STGG from each specimen was transported on dry ice to the CDC Meningitis Laboratory, where DNA extractions and rt-PCR were performed.

3. A total of 33 NP swabs and 35 nasal washes (NWs) were taken from 24 participants ages 21–57 years during ≤7 visits in a Spring 2009 study conducted in the NIHR Biomedical Research Centre in Microbial Diseases at the Liverpool School of Tropical Medicine, Liverpool, United Kingdom (UK). Swab specimens were collected as previously described [25] with some modifications and placed directly into 1 ml STGG [23], then transported to the laboratory on wet ice for culture and processing. 900 µl of each specimen was frozen at −80°C for subsequent DNA extraction and rt-PCR in Liverpool.

NWs were collected as previously described [26] and the nasal fluid from both nostrils of each study participant was pooled. NWs were collected in this manner from each participant. Once at the lab, NW specimens were centrifuged at 1509× g for seven minutes and the pellet was re-suspended in 1 ml STGG. The processing of the NWs from this point was the same as that for the NP specimens.

### DNA preparation and quantification

Genomic DNA was prepared for use in the various steps of assay design and optimization using the QIAamp DNA Mini Kit (QIAGEN, Valencia, California) using Protocol C then quantified for use in standard curve experiments using a NanoDrop ND-1000 or 8000 spectrophotometer (Nanodrop Technologies, Wilmington, Delaware). Preparation of DNA from bacterial isolates was performed as previously described [5].

For CSF specimens and the Brazilian NP swab eluates, DNA extraction was conducted as previously described for clinical specimens [23]. DNA extractions were performed on the CHOA carriage study NP swab eluates using the MagNA Pure LC instrument and the DNA Isolation Kit III (Bacteria, Fungi) per the manufacturer's instructions (Roche Diagnostics GmbH, Mannheim, Germany). DNA was extracted from the UK NP and NW specimens using the QIAsymphony SP System and the QIAsymphony Virus/Bacteria Midi Kit (QIAGEN Inc., UK) according to the manufacturer's instructions. All extracted DNA was stored at −20°C.

### Gene sequencing

The *sodC* sequencing templates were prepared by conventional PCR using Expand High Fidelity Enzyme Mix (Roche Diagnostics GmbH) per the manufacturer's instructions and primers (see Table 4) that were designed based on a consensus of *sodC* from meningococcal strains Z2491 (nts 1521721–1522258), FAM18, and MC58 (respective GenBank accession numbers AL157959.1, AM421808.1, and AE002098.2) [19]. DNA sequencing was performed using the BigDye Terminator v3.1 Cycle Sequencing Kit (Applied Biosystems, Foster City, California) and an ABI PRISM® 3130*xl* Genetic Analyzer (Applied Biosystems) and a consensus sequence was generated using Lasergene DNAStar v. 7 Program SeqMan.

**Table 4:** Primers and probe used in this study.

Target Gene	Oligo Designation	Use	5′ to3′ Nucleotide Sequence	Amplicon Size (bp)	Final Concentration (nM)
*sodC*	Nm *sodC*-Fwd	PCR, sequencing	CCT TAT TAG CAC TAG CGG TTA G		400, 160
	Nm *sodC*-Rev	PCR, sequencing	CCG GTC ATC TTT TAT GCT CCA A	537	400, 160
*sodC*	Nm *sodC* Fwd 351	rt-PCR	GCA CAC TTA GGT GAT TTA CCT GCA T		300
	Nm *sodC* Rev 478	rt-PCR	CCA CCC GTG TGG ATC ATA ATA GA	127	600
	Nm *sodC* Pb387	rt-PCR	(FAM)-CAT GAT GGC ACA GCA ACA AAT CCT GTT T-(BHQ1)		100

### rt-PCR primer and probe design

The *sodC* consensus sequence was entered into Primer Express 3.0 (Applied Biosystems). Primers and probes were analyzed for homology to other known sequences using the Basic Local Alignment Search Tool (BLAST) [27]. Primers were tested for optimal concentration in triplicate or quadruplicate in combinations of final concentrations of 100, 300, 600, and 900 nM; the probe was tested in triplicate at final concentrations of 50, 100, 200, and 300 nM. The amplified product is located at nt 1427446 in MC58 (GenBank accession number AE002098.2).

### rt-PCR

A Stratagene Mx3005P (Agilent, La Jolla, California) and QuantiTect SYBR Green Master Mix (QIAGEN) were used to optimize primer concentrations. Cycle parameters were 2 minutes at 50°C, 10 minutes at 95°C, and then 50× (15 seconds at 95°C plus 1 minute at 60°C). Product dissociation curves were generated using one round of the following cycle parameters at the end of the primer optimization run: one minute at 95°C, 30 seconds at 55°C, and 30 seconds at 95°C. Master mixes contained 4.5 µl sterile PCR grade water (Roche Diagnostics), 12.5 µl TaqMan®2× PCR Master Mix (Applied Biosystems), 300 nM forward primer, 600 nM reverse primer, 100 nM probe, and 2 µl template DNA per total reaction volume of 25 µl. With each reaction plate that was run, cell lysates from known Nm served as positive external amplification controls, while no-template controls (NTCs) served as negative external amplification controls.

rt-PCR detection of *ctrA* in the MD isolates [20] was performed at the Laboratory of the Maryland Department of Health and Mental Hygiene using an Applied Biosystems 7500 or 7700 rt-PCR System (Applied Biosystems, Inc., Foster City, CA).

A Corbett Rotor-Gene 6000 (QIAGEN) and Corbett software 24 series 1.7 were employed in the UK for rt-PCR and data analysis on extractions of NWs and NP eluates. In every UK *sodC* reaction, 2.5 µl of extracted DNA was used.

Throughout this study, C_t_ values≤35 were considered positive; C_t_ values in the range of 36–40 equivocal; and C_t_ values>40 negative. Extracted DNA from equivocal clinical specimens was diluted 1∶4 and 1∶10 in an attempt to dilute possible inhibitors of the reaction. Dilutions were then re-tested in duplicate. If the average C_t_ of the diluted specimen fell below 35, that specimen was considered positive. If the average C_t_ of the diluted specimen remained in the 35–40 range, that specimen was considered negative.

### MLST

MLST was performed as described by Maiden et al. [28]. Sequences were assembled and alleles were determined using Staden [29] v. 1.5.3 and Sequence Typing Analysis Retrieval System (STARS) v. 1.2a or the Meningococcus Genome Informatics Platform (MGIP) (http://mgip.biology.gatech.edu.) [30]. Sequence types (STs) and clonal complexes were assigned by querying http://pubmlst.org/neisseria or http://neisseria.org/nm/typing.

### Nucleotide sequence accession numbers

The GenBank accession numbers for the Nm *sodC* sequences generated in this study are listed in Table S3.

## Results

### Primer and probe design

A consensus nucleotide sequence was built based on 2× coverage of *sodC* sequenced from each of 14 geographically and temporally dispersed groupable and NG meningococci (Table S1). Over the 439–473 out of 560 *sodC* nucleotides sequenced in these isolates, these meningococcal isolates were 99–100% identical to each other and 81% identical to an Hi *sodC* consensus that was built using two sequences from GenBank (accession numbers M84012 and AF549211). Given that *sodC* was likely acquired by Nm via horizontal transfer from Hi [18], primers and a probe that differed by at least three nucleotides per oligo between Nm and Hi *sodC* were chosen (Figure S1, Table 4).

BLAST results showed that the primers had no homology over 78% nucleotide identity with any genes but meningococcal *sodC*. The only notable homology found for the probe was a two-nucleotide difference with *H. parainfluenzae sodC*; the primers, however, showed no homology to this gene.

### Lower limit of detection (LLD)

Standard curves were generated by testing genomic DNA from four invasive meningococcal isolates with the *sodC* assay (data not shown). LLDs at a C_t_ value of 35 were found to be 39, 70, 101, and 82 genomes per reaction, yielding an average of 73 genomes detected per reaction. The average reaction efficiency was 100% and the average R^2^ value was 0.9925.

### Sensitivity of the *sodC* assay

Invasive (i.e., isolated from CSF, blood, joint fluid, or autopsy tissue, n = 76) and non-invasive (i.e., including carriage isolates and those from sputum or oral swab specimens, n = 30) known Nm isolates from the CDC Meningitis Laboratory Strain Collection were tested with the *sodC* assay. All isolates were positive for *sodC*, including 26 *ctrA*-negative NG isolates, with a median C_t_ value of 19.7, mean of 19.9±1.9, and range of 16.4 to 26.0 (Table 1 and Table S2).

518/520 (99.6%) of meningococcal carriage isolates from two U.S. carriage studies [20], [21] were positive for *sodC* (median C_t_ 16.9, mean 17.0±1.5, and range 13.5 to 29.3), while *ctrA* detected only 368/520 (70.8%) of these isolates (median C_t_ 19.0, mean 19.2±2.7, and range 13.5 to 34.0) (Table 1 and Table S2). The two *sodC*-negative carried Nm were SASG NG, *ctrA*-negative but were confirmed to be Nm ST-1117 and ST-4788, both cc1117; both were isolated from the same study participant at different time points. Therefore, 176/178 (98.9%) SASG NG, *ctrA*-negative invasive and non-invasive Nm isolates were *sodC*-positive. Four *sodC*-negative carriage study isolates that were identified as Nm by conventional methods were re-investigated and shown to actually be non-Nm.

### Specificity of the *sodC* assay

None of 35 non-Nm from various sources and none of 209 non-meningococcal carriage study [20], [24] isolates were detected by *sodC* (100% specificity) (Table 2). Interestingly, *sodC* identified one isolate, M16160, as Nm when other standard carriage study tests could not correctly resolve its species. It was originally identified as *N. polysaccharea*/*N.* spp. by NH strip, but upon re-investigation, did not grow at room temperature on chocolate agar, indicating that it is not *N. polysaccharea* [1]. All 7 meningococcal housekeeping genes were readily amplified during MLST, suggesting that M16160 is indeed Nm (ST-7456, cc60), and again showing *sodC* to be a useful tool for definitive identification of carriage isolates.

### Detection of Nm in clinical specimens

The ability of *sodC* to detect Nm was assessed and was compared to *ctrA* as the target gene [5] using extracted DNA from 120 Turkish CSF specimens and 37 U.S. clinical specimens. *ctrA* detected Nm in 21/157 (13.4%) specimens (20 CSF and 1 blood), while *sodC* was positive for those 21 plus four additional CSF specimens (25/157, 15.9%) (Table 3 and Table S4). Therefore, *sodC* was 100% (95% confidence interval [CI]: 84–100%) sensitive compared to *ctrA* at detection of Nm from these clinical specimens, but specificity is difficult to calculate using *ctrA* as a reference test. The C_t_ values for the four *ctrA*-negative, *sodC*-positive CSF extractions averaged 40.4 for *ctrA* while their *sodC* C_t_ values averaged 32.0.

One *ctrA*-negative, *sodC*-positive CSF specimen was Nm culture-positive (1/157, 0.6%); the remaining specimens were either culture-negative (139/157) or culture was not attempted or not reported (17/157). Therefore, *sodC* was 88% (95% CI: 82–93%) specific compared to culture at detection of Nm from the clinical specimens for which culture was attempted (Table S4), but sensitivity is undetermined due to the low number of culture-positives.

### Detection of Nm in carriage specimens

Results from all three carriage studies are summarized in Table 5. None (0/223, 0%) of the Brazilian NP swab eluate extractions tested at CDC were *ctrA*-positive while 3 (3/223, 1.3%) were *sodC*-positive, yielding C_t_ values of 29.1, 26.2, and 25.5. The *ctrA*-negative, *sodC*-positive specimens were negative for serogroups A, B, C, W135, X, and Y by rt-PCR. In the 1/3 *ctrA*-negative, *sodC*-positive specimen that had a sufficient amount of extraction volume remaining, MLST confirmed the presence of meningococcal DNA (ST-823, cc198). All 23 Hi culture-positive Brazilian specimens were *ctrA*- and *sodC*-negative.

**Table 5:** Carriage specimens tested for *sodC* and *ctrA* and compared to culture.

Specimen	Total n	n *sodC* ^+^	% sodC^+^	95% CI^1^	n *ctrA* ^+^	% ctrA^+^	95% CI	n Nm Culture Positive	% Culture Positive	95% CI
NP swab eluate	547	3	0.5	0% to 2%	1^2^	0.2	0% to 1%	0	0.0	0% to 1%
Nasal wash	35	2^3^	5.7	1% to 19%	3	8.6	2% to 23%	1^4^	2.9	0% to 15%
Total	582	5	0.9	n/a^5^	4	0.7	n/a	1	0.2	n/a

1 Exact binomial 95% confidence interval.

2 This *ctrA*-positive NP swab eluate was *sodC*-negative.

3 Both of these *sodC*-positive NP swab eluates were *ctrA*-positive.

4 This Nm culture-positive NW was *ctrA*-positive, *sodC*-positive.

5 n/a, not applicable.

All (291/291, 100%) NP swab eluate extractions from the Georgia CHOA carriage study were *ctrA*-negative and *sodC*-negative, as expected, since no Nm was cultured. These specimens were, however, culture-positive for *N.* spp. (n = 3), *N. polysaccharea* (n = 1), *M. catarrhalis* (n = 8), *H. parainfluenzae* (n = 9), *H. haemolyticus* (n = 1), and Hi (n = 76), further demonstrating the specificity of *sodC*.

*S. aureus* (n = 17), alpha-hemolytic streptococci (n = 18), *M. catarrhalis* (n = 5), diptheroids (n = 10), *N. polysaccharea* (n = 1), *N. cinerea* (n = 2), and *N. meningitidis* (n = 1) were cultured from the 68 UK carriage study specimens; the Nm culture-positive NW was *ctrA*-positive and *sodC*-positive. *sodC* and *ctrA* were negative for all of the non-Nm culture-positive specimens except 1 *ctrA*-positive, *sodC*-positive NW that grew *N. cinerea* and alpha-hemolytic streptococci. 1/33 (3%) NP swab eluate extractions from the UK carriage study was *ctrA*-positive, 0/33 were *sodC*-positive, and 0/33 were Nm culture-positive. 3/35 (8.6%) NW extractions were *ctrA*-positive, 2/35 (5.7%) were also *sodC*-positive; of these, one (1/35, 2.9%) *ctrA*-positive, *sodC*-positive NW was Nm culture-positive. The *ctrA*-positive, *sod*C-negative NP specimen (average *ctrA* C_t_ 35.0±0.3, average *sodC* C_t_ 37.8±0.8) and the *ctrA*-positive, *sodC*-negative NW (average *ctrA* C_t_ value of 34.3±0.2 and an average *sodC* C_t_ value of 35.1±0.2) were both from patient 8, visit 3; this patient had the Nm-positive culture at visit 1 and the *N. cinerea*-positive culture at visit 2.

## Discussion

Outbreaks of meningococcal disease occur in the U.S. and meningococcal disease continues to be a leading cause of bacterial meningitis worldwide. Regardless of location, when outbreaks of meningococcal disease occur or new vaccines are introduced, carriage studies are often performed to answer epidemiologic and biological questions (e.g., extent of the disease-causing clone circulating in the population) or demonstrate vaccine effects on carriage and therefore its potential impact on transmission and herd immunity.

Culture detection of Nm is 100% specific and should be attempted if possible, but it is limited by low sensitivity [31], [32] and 24- to 72-hour incubation periods. The rapidity, sensitivity, and specificity of rt-PCR are rendering this technique an increasingly employed Nm detection method in clinical and reference laboratories [33]–[38]. Many laboratories use *ctrA* as a gene target for rt-PCR detection of Nm [3], [5], [10], [35], [38], [39]. However, given that *ctrA* is not present in 16% or more of carriage isolates [11], [12], it is not a suitable target gene for PCR on carriage study specimens. The reliability of *ctrA*-based PCR assays in detecting NG invasive Nm isolates [35] or specimens containing them, although rare, is also called into question by what is now known about the genetics of nongroupability in Nm carriage isolates [11], [12] and the well-described genome plasticity of this naturally competent nasopharyngeal resident. Indeed, *ctrA*-based rt-PCR has recently been reported to generate false-negative results due to sequence variations in *ctrA* [40].

The *sodC* assay was designed to detect Nm, especially among carriage isolates or specimens that lack *ctrA*, and it did, detecting 98.9% of carried and invasive *ctrA*-negative NG Nm isolates and from four *ctrA*-negative clinical and three *ctrA*-negative carriage specimens. *sodC* also surpassed culture in sensitivity by detecting Nm in 24 culture-negative clinical specimens and in at least one Nm culture-negative carriage specimen. The assay significantly improves specificity over culture, but sensitivity of *sodC* compared to culture is difficult to determine due to the lack of culture-positive clinical specimens received. This limitation underscores the benefit that the *sodC* real-time PCR assay will provide for the detection of Nm, especially in developing countries. Additionally, the specificity of *sodC* compared to *ctrA* cannot accurately be determined; the literature does not support the idea that *ctrA* is the ideal “gold standard” that is needed for a comparison of specificity [11], [12], [40]. *sodC* detects 73 genomes per 25-µl rt-PCR reaction, well within the range (≤10^3^–10^5^ CFU/ml) that would be found in CSF [9].

None of the 88 Hi isolates (Table 2) or the 100 Hi culture-positive Brazilian and CHOA carriage specimens tested were detected by this Nm *sodC* assay. *sodC* is also found in *H*. spp. other than Hi [41]–[43], but none of the 15 non-Hi *H*. spp. isolates (Table 2) and none of the 10 non-Hi *H*. spp. culture-positive CHOA carriage specimens tested were detected by *sodC*. To our knowledge, *sodC* has not been found in any other *Neisseria* spp. besides the meningococcus [17], [18], [44]. The species specificity of *sodC* was supported by the 100% specificity of the *sodC* assay when tested against a panel of 244 non-Nm isolates, including 107 *N*. spp. isolates, and by 100% specificity of the assay when tested on carriage specimens, including 8 UK and CHOA specimens that were culture-positive for non-Nm *N*. spp.

The C_t_ value interpretation cutoffs and thermocycler parameters used for the *sodC* assay are the same as those used for detection of Nm serogroups A, B, C, W135, X, and Y [5] and for detection of Hi *hpd* [45] and *S. pneumoniae lytA* [23], making it convenient to perform multiple assays on isolates or specimens in one 96-well plate.

In summary, an extremely rapid, sensitive, and specific rt-PCR assay was developed and validated for the detection of meningococcal isolates and Nm from clinical and carriage specimens. This new diagnostic tool will be especially useful for confirming isolate species identification and detecting meningococci from carriage and clinical specimens, regardless of the organism's encapsulation status.

## Supporting Information

Figure S1 Nm *sodC* compared to Hi *sodC* and primer and probe location. *sodC* was sequenced from 14 groupable and NG meningococci and a consensus nucleotide sequence was generated. An *H. influenzae sodC* consensus was built using two sequences from GenBank (accession numbers M84012 and AF549211). Nucleotide differences from the meningococcal sequence are white letters highlighted in black in the *Haemophilus* sequence. The primers and probe (highlighted in gray) were chosen at positions not only containing at least three nucleotide differences between Nm and Hi, but also where *sodC* nucleotide sequence was conserved among the meningococcal isolates that were sequenced. The meningococcal *sodC* open reading frame is 560 nucleotides long; the portion of the gene depicted corresponds to nucleotides 255–513.
(DOCX)

Table S1 Nm control isolates used for *sodC* assay design and optimization.
(DOCX)

Table S2 626 Nm isolates used to test the sensitivity of the *sodC* assay.
(DOCX)

Table S3 Nm *sodC* sequences generated in this study.
(DOCX)

Table S4 Two-by-two contingency tables that were the basis for the calculation of *sodC* PCR sensitivity and specificity compared to culture and *ctrA* PCR. (a) Comparison of *sodC* PCR to culture for 140 clinical specimens. (b) Comparison of *sodC* PCR to *ctrA* PCR for 157 clinical specimens.
(DOCX)
