# Supplementary material for: sodC-Based Real-Time PCR for Detection of Neisseria meningitidis
Source: PLoS One. 2011 May 5;6(5):e19361. doi: 10.1371/journal.pone.0019361 (PMC3088665; doi:10.1371/journal.pone.0019361)
Supplement: Figure S1 — Nm sodC compared to Hi sodC and primer and probe location. sodC was sequenced from 14 groupable and NG meningococci and a consensus nucleotide sequence was generated. An H. influenzae sodC consensus was built using two sequences from GenBank (accession numbers M84012 and AF549211). Nucleotide differences from the meningococcal sequence are white letters highlighted in black in the Haemophilus sequence. The primers and probe (highlighted in gray) were chosen at positions not only containing at least three nucleotide differences between Nm and Hi, but also where sodC nucleotide sequence was conserved among the meningococcal isolates that were sequenced. The meningococcal sodC open reading frame is 560 nucleotides long; the portion of the gene depicted corresponds to nucleotides 255–513. (DOCX) [file pone.0019361.s001.docx]

**Figure S1. Nm *sodC* compared to Hi *sodC* and primer and probe location.** *sodC* was sequenced from 14 groupable and NG meningococci and a consensus nucleotide sequence was generated. An *H. influenzae* *sodC* consensus was built using two sequences from GenBank (accession numbers M84012 and AF549211). Nucleotide differences from the meningococcal sequence are white letters highlighted in black in the *Haemophilus* sequence. The primers and probe (highlighted in gray) were chosen at positions not only containing at least three nucleotide differences between Nm and Hi*,* but also where *sodC* nucleotide sequence was conserved among the meningococcal isolates that were sequenced. The meningococcal *sodC* open reading frame is 560 nucleotides long; the portion of the gene depicted corresponds to nucleotides 255-513.

Supplementary Data Figure S1
