# Supplementary material for: sodC-Based Real-Time PCR for Detection of Neisseria meningitidis
Source: PLoS One. 2011 May 5;6(5):e19361. doi: 10.1371/journal.pone.0019361 (PMC3088665; doi:10.1371/journal.pone.0019361)
Supplement: Table S1 — Nm control isolates used for sodC assay design and optimization. (DOCX) [file pone.0019361.s002.docx]

Table S1. Nm Control Isolates Used for *sodC* Assay Design and Optimization
