# Supplementary material for: sodC-Based Real-Time PCR for Detection of Neisseria meningitidis
Source: PLoS One. 2011 May 5;6(5):e19361. doi: 10.1371/journal.pone.0019361 (PMC3088665; doi:10.1371/journal.pone.0019361)
Supplement: Table S2 — 626 Nm isolates used to test the sensitivity of the sodC assay. (DOCX) [file pone.0019361.s003.docx]

Table S2. 626 Nm isolates used to test the sensitivity of the *sodC* assay.

| Strain Name | SASG | SG-PCR^1^ | Study or Sender Location | Invasive or Carriage | | *ctrA* | *ctrA* C_t_^2^ | *sodC* | Avg *sodC* C_t_^3^ | Additional Molecular Tests Confirming ID as Nm | |
| --- | --- | --- | --- | --- | --- | --- | --- | --- | --- | --- | --- |
| M04937 | NG | nd^4^ | Kellerman | Carriage | | - | No C_t_ | + | 19.4 | ET-993^5^ , ST-198^6^ | |
| M04951 | NG | nd | Kellerman | Carriage | | - | No C_t_ | + | 17.8 | ET-1203, ST-2056 | |
| M04953 | NG | nd | Kellerman | Carriage | | - | No C_t_ | + | 19.6 | ET-1205, ST-198 | |
| M04955 | NG | nd | Kellerman | Carriage | | - | No C_t_ | + | 20.5 | ET-1207, ST-2051 | |
| M04959 | NG | nd | Kellerman | Carriage | | - | No C_t_ | + | 18.0 | ET-1211, ST-198 | |
| M04986 | NG | nd | Kellerman | Carriage | | - | No C_t_ | + | 17.3 | ET-1213, LH^7^ | |
| M04990 | NG | nd | Kellerman | Carriage | | - | No C_t_ | + | 18.4 | ET-1217, ST-2049 | |
| M05013 | NG | nd | Kellerman | Carriage | | - | No C_t_ | + | 17.6 | ET-1125, LH | |
| M05015 | NG | nd | Kellerman | Carriage | | - | No C_t_ | + | 15.2 | ET-1218, LH | |
| M05019 | NG | nd | Kellerman | Carriage | | - | No C_t_ | + | 16.0 | ET-1222, ST-2047 | |
| M05020 | NG | nd | Kellerman | Carriage | | - | No C_t_ | + | 17.1 | ET-1223, ST-198 | |
| M05021 | NG | nd | Kellerman | Carriage | | - | No C_t_ | + | 16.1 | ET-1223, ST-198 | |
| M05034 | NG | nd | Kellerman | Carriage | | - | No C_t_ | + | 16.0 | ET-1223, LH | |
| M05037 | NG | nd | Kellerman | Carriage | | - | No C_t_ | + | 16.8 | ET-1223, LH | |
| M05042 | NG | nd | Kellerman | Carriage | | - | No C_t_ | + | 15.3 | ET-1229, LH | |
| M05048 | NG | nd | Kellerman | Carriage | | - | No C_t_ | + | 14.5 | ET-1229, LH | |
| M05060 | NG | nd | Kellerman | Carriage | | - | No C_t_ | + | 15.2 | ET-1133, LH | |
| M05071 | NG | nd | Kellerman | Carriage | | - | No C_t_ | + | 13.8 | ET-1137, LH | |
| M05075 | NG | nd | Kellerman | Carriage | | - | No C_t_ | + | 14.5 | ET-1238, ST-2050 | |
| M05078 | NG | nd | Kellerman | Carriage | | - | No C_t_ | + | 14.3 | ET-1239, LH | |
| M05082 | NG | nd | Kellerman | Carriage | | - | No C_t_ | + | 15.3 | ET-1241, LH | |
| M05085 | NG | nd | Kellerman | Carriage | | - | No C_t_ | + | 19.7 | ET-1142, LH | |
| M05105 | NG | nd | Kellerman | Carriage | | - | No C_t_ | + | 14.4 | ET-1245, LH | |
| M05106 | NG | nd | Kellerman | Carriage | | - | No C_t_ | + | 16.8 | ET-1246, LH | |
| M05046 | NG | nd | Kellerman | Carriage | | - | 36.0 | + | 16.9 | ET-1223, LH | |
| M04936 | NG | nd | Kellerman | Carriage | | + | 19.8 | + | 18.6 | ET-992, ST-103 | |
| M04941 | NG | nd | Kellerman | Carriage | | + | 15.0 | + | 29.3 | ET-995, ST-60 | |
| M04943 | NG | nd | Kellerman | Carriage | | + | 21.2 | + | 19.6 | ET-996, ST-23 | |
| M04944 | NG | nd | Kellerman | Carriage | | + | 15.6 | + | 19.4 | ET-997, ST-1975 | |
| M04945 | NG | nd | Kellerman | Carriage | | + | 19.4 | + | 18.2 | ET-998, ST-103 | |
| M04946 | NG | nd | Kellerman | Carriage | | + | 20.9 | + | 18.6 | ET-999, ST-23 | |
| M04947 | NG | nd | Kellerman | Carriage | | + | 19.8 | + | 18.1 | ET-962, ST-2053 | |
| M04948 | NG | nd | Kellerman | Carriage | | + | 20.8 | + | 18.5 | ET-1200, LH | |
| M04949 | NG | nd | Kellerman | Carriage | | + | 18.6 | + | 17.2 | ET-1201, ST-2052 | |
| M04950 | NG | nd | Kellerman | Carriage | | + | 14.9 | + | 15.9 | ET-1202, ST-103 | |
| M04952 | NG | nd | Kellerman | Carriage | | + | 21.6 | + | 19.9 | ET-1204, ST-790 | |
| M04954 | NG | nd | Kellerman | Carriage | | + | 18.5 | + | 18.3 | ET-1206, ST-136 | |
| M04956 | NG | nd | Kellerman | Carriage | | + | 19.8 | + | 18.8 | ET-1208, ST-2054 | |
| M04957 | NG | nd | Kellerman | Carriage | | + | 20.8 | + | 15.3 | ET-1209, ST-812 | |
| M04958 | NG | nd | Kellerman | Carriage | | + | 19.6 | + | 18.6 | ET-1210, ST-178 | |
| M04985 | NG | nd | Kellerman | Carriage | | + | 21.2 | + | 18.0 | ET-1212, LH | |
| M04987 | NG | nd | Kellerman | Carriage | | + | 20.5 | + | 17.7 | ET-1214, LH | |
| M04988 | NG | nd | Kellerman | Carriage | | + | 22.9 | + | 19.2 | ET-1215, LH | |
| M04989 | NG | nd | Kellerman | Carriage | | + | 21.0 | + | 17.7 | ET-1216, ST-23 | |
| M05016 | NG | nd | Kellerman | Carriage | | + | 15.7 | + | 15.6 | ET-1219, LH | |
| M05017 | NG | nd | Kellerman | Carriage | | + | 15.8 | + | 15.4 | ET-1220, LH | |
| M05018 | NG | nd | Kellerman | Carriage | | + | 20.6 | + | 16.5 | ET-1221, ST-790 | |
| M05033 | NG | nd | Kellerman | Carriage | | + | 19.3 | + | 16.2 | ET-1224, LH | |
| M05035 | NG | nd | Kellerman | Carriage | | + | 20.7 | + | 17.3 | ET-1225, LH | |
| M05036 | NG | nd | Kellerman | Carriage | | + | 21.6 | + | 18.4 | ET-1221, LH | |
| M05038 | NG | nd | Kellerman | Carriage | | + | 22.6 | + | 18.5 | ET-1221, LH | |
| M05039 | NG | nd | Kellerman | Carriage | | + | 20.0 | + | 17.3 | ET-1226, LH | |
| M05040 | NG | nd | Kellerman | Carriage | | + | 19.5 | + | 16.6 | ET-1227, LH | |
| M05041 | NG | nd | Kellerman | Carriage | | + | 19.4 | + | 15.1 | ET-1228, LH | |
| M05047 | NG | nd | Kellerman | Carriage | | + | 18.5 | + | 15.8 | ET-1221, LH | |
| M05049 | NG | nd | Kellerman | Carriage | | + | 19.7 | + | 16.3 | ET-1231, LH | |
| M05050 | NG | nd | Kellerman | Carriage | | + | 18.2 | + | 14.0 | ET-1232, LH | |
| M05051 | NG | nd | Kellerman | Carriage | | + | 18.1 | + | 14.4 | ET-1233, LH | |
| M05052 | NG | nd | Kellerman | Carriage | | + | 19.8 | + | 16.8 | ET-1234, LH | |
| M05053 | NG | nd | Kellerman | Carriage | | + | 18.7 | + | 15.0 | ET-1235, LH | |
| M05054 | NG | nd | Kellerman | Carriage | | + | 19.7 | + | 15.0 | ET-1236, ST-54 | |
| M05073 | NG | nd | Kellerman | Carriage | | + | 18.1 | + | 14.2 | ET-1120, LH | |
| M05074 | NG | nd | Kellerman | Carriage | | + | 19.0 | + | 15.2 | ET-1237, ST-2048 | |
| M05076 | NG | nd | Kellerman | Carriage | | + | 20.2 | + | 15.1 | ET-508, LH | |
| M05077 | NG | nd | Kellerman | Carriage | | + | 21.2 | + | 14.8 | ET-508, LH | |
| M05079 | NG | nd | Kellerman | Carriage | | + | 20.5 | + | 15.3 | ET-1240, LH | |
| M05080 | NG | nd | Kellerman | Carriage | | + | 21.6 | + | 16.2 | ET-508, LH | |
| M05081 | NG | nd | Kellerman | Carriage | | + | 19.5 | + | 15.6 | ET-527, LH | |
| M05096 | NG | nd | Kellerman | Carriage | | + | 22.6 | + | 17.2 | ET-508, LH | |
| M05097 | NG | nd | Kellerman | Carriage | | + | 20.5 | + | 16.4 | ET-1242, LH | |
| M05098 | NG | nd | Kellerman | Carriage | | + | 20.4 | + | 16.3 | ET-1243, LH | |
| M05099 | NG | nd | Kellerman | Carriage | | + | 21.3 | + | 17.3 | ET-1243, LH | |
| M05100 | NG | nd | Kellerman | Carriage | | + | 19.4 | + | 16.0 | ET-1243, LH | |
| M05101 | NG | nd | Kellerman | Carriage | | + | 19.3 | + | 14.9 | ET-1244, LH | |
| M05102 | NG | nd | Kellerman | Carriage | | + | 18.5 | + | 14.8 | ET-1240, LH | |
| M05103 | NG | nd | Kellerman | Carriage | | + | 20.6 | + | 16.0 | ET-508, LH | |
| M05104 | NG | nd | Kellerman | Carriage | | + | 18.4 | + | 14.6 | ET-1242, LH | |
| M05107 | NG | nd | Kellerman | Carriage | | + | 19.9 | + | 15.4 | ET-1247, LH | |
| M05108 | NG | nd | Kellerman | Carriage | | + | 19.8 | + | 15.4 | ET-1248, LH | |
| M05109 | NG | nd | Kellerman | Carriage | | + | 19.9 | + | 14.5 | ET-1249, LH | |
| M05110 | NG | nd | Kellerman | Carriage | | + | 22.0 | + | 14.9 | ET-1250, LH | |
| M04915 | B | nd | Kellerman | Carriage | | + | 22.3 | + | 19.1 | ET-1101, LH | |
| M04916 | B | nd | Kellerman | Carriage | | + | 21.5 | + | 18.6 | ET-1104, LH | |
| M04917 | B | nd | Kellerman | Carriage | | + | 21.3 | + | 19.0 | ET-1105, LH | |
| M04924 | B | nd | Kellerman | Carriage | | + | 20.5 | + | 16.7 | ET-1099, LH | |
| M04925 | B | nd | Kellerman | Carriage | | + | 20.6 | + | 17.6 | ET-1106, LH | |
| M04926 | B | nd | Kellerman | Carriage | | + | 22.4 | + | 19.2 | ET-1107, LH | |
| M04939 | B | nd | Kellerman | Carriage | | + | 21.4 | + | 18.6 | ET-1110, LH | |
| M04940 | B | nd | Kellerman | Carriage | | + | 21.4 | + | 18.0 | ET-1111, LH | |
| M04942 | B | nd | Kellerman | Carriage | | + | 20.7 | + | 17.2 | ET-1112, LH | |
| M04982 | B | nd | Kellerman | Carriage | | + | 20.0 | + | 17.2 | ET-1117, LH | |
| M04983 | B | nd | Kellerman | Carriage | | + | 19.3 | + | 16.5 | ET-1118, LH | |
| M04984 | B | nd | Kellerman | Carriage | | + | 20.1 | + | 17.6 | ET-1119, LH | |
| M05006 | B | nd | Kellerman | Carriage | | + | 19.6 | + | 15.4 | ET-1120, LH | |
| M05007 | B | nd | Kellerman | Carriage | | + | 22.0 | + | 16.3 | ET-1120, LH | |
| M05008 | B | nd | Kellerman | Carriage | | + | 22.6 | + | 16.7 | ET-1121, LH | |
| M05009 | B | nd | Kellerman | Carriage | | + | 21.2 | + | 16.0 | ET-1120, LH | |
| M05010 | B | nd | Kellerman | Carriage | | + | 22.2 | + | 16.5 | ET-1122, LH | |
| M05011 | B | nd | Kellerman | Carriage | | + | 22.6 | + | 17.4 | ET-1123, LH | |
| M05012 | B | nd | Kellerman | Carriage | | + | 19.7 | + | 15.8 | ET-1124, LH | |
| M05030 | B | nd | Kellerman | Carriage | | + | 21.3 | + | 16.6 | ET-1126, LH | |
| M05031 | B | nd | Kellerman | Carriage | | + | 20.9 | + | 16.2 | ET-1127, LH | |
| M05044 | B | nd | Kellerman | Carriage | | + | 20.8 | + | 16.7 | ET-1128, LH | |
| M05055 | B | nd | Kellerman | Carriage | | + | 19.6 | + | 15.4 | ET-1129, LH | |
| M05056 | B | nd | Kellerman | Carriage | | + | 20.6 | + | 15.5 | ET-1130, LH | |
| M05057 | B | nd | Kellerman | Carriage | | + | 22.9 | + | 16.0 | ET-1131, LH | |
| M05058 | B | nd | Kellerman | Carriage | | + | 22.0 | + | 15.3 | ET-1130, LH | |
| M05059 | B | nd | Kellerman | Carriage | | + | 22.2 | + | 15.4 | ET-1132, LH | |
| M05061 | B | nd | Kellerman | Carriage | | + | 21.2 | + | 15.3 | ET-1134, LH | |
| M05069 | B | nd | Kellerman | Carriage | | + | 21.3 | + | 14.3 | ET-1135, LH | |
| M05070 | B | nd | Kellerman | Carriage | | + | 21.2 | + | 15.7 | ET-1136, LH | |
| M05083 | B | nd | Kellerman | Carriage | | + | 22.4 | + | 16.4 | ET-1141, LH | |
| M05084 | B | nd | Kellerman | Carriage | | + | 19.2 | + | 14.0 | ET-1141, LH | |
| M05086 | B | nd | Kellerman | Carriage | | + | 21.8 | + | 15.3 | ET-1141, LH | |
| M05045 | C | nd | Kellerman | Carriage | | + | 20.0 | + | 15.3 | ET-24, LH | |
| M05072 | C | nd | Kellerman | Carriage | | + | 22.7 | + | 14.6 | ET-234, LH | |
| M04918 | Y | nd | Kellerman | Carriage | | + | 20.9 | + | 17.5 | ET-830, LH | |
| M04919 | Y | nd | Kellerman | Carriage | | + | 23.3 | + | 19.6 | ET-831, LH | |
| M04920 | Y | nd | Kellerman | Carriage | | + | 22.8 | + | 18.6 | ET-830, LH | |
| M04921 | Y | nd | Kellerman | Carriage | | + | 22.8 | + | 19.6 | ET-830, LH | |
| M04922 | Y | nd | Kellerman | Carriage | | + | 23.3 | + | 19.8 | ET-830, LH | |
| M04927 | Y | nd | Kellerman | Carriage | | + | 22.6 | + | 19.2 | ET-508, LH | |
| M04928 | Y | nd | Kellerman | Carriage | | + | 22.3 | + | 19.8 | ET-508, LH | |
| M04929 | Y | nd | Kellerman | Carriage | | + | 21.3 | + | 18.0 | ET-516, LH | |
| M04930 | Y | nd | Kellerman | Carriage | | + | 21.4 | + | 18.5 | ET-516, LH | |
| M04931 | Y | nd | Kellerman | Carriage | | + | 21.6 | + | 18.1 | ET-516, LH | |
| M04932 | Y | nd | Kellerman | Carriage | | + | 24.1 | + | 19.8 | ET-508, LH | |
| M04933 | Y | nd | Kellerman | Carriage | | + | 21.3 | + | 19.1 | ET-516, LH | |
| M04934 | Y | nd | Kellerman | Carriage | | + | 22.7 | + | 19.4 | ET-838, LH | |
| M04935 | Y | nd | Kellerman | Carriage | | + | 21.3 | + | 18.6 | ET-835, LH | |
| M04960 | Y | nd | Kellerman | Carriage | | + | 24.7 | + | 19.6 | ET-508, LH | |
| M04961 | Y | nd | Kellerman | Carriage | | + | 23.6 | + | 18.7 | ET-508, LH | |
| M04962 | Y | nd | Kellerman | Carriage | | + | 23.3 | + | 17.6 | ET-508, LH | |
| M04963 | Y | nd | Kellerman | Carriage | | + | 23.7 | + | 18.8 | ET-508, LH | |
| M04964 | Y | nd | Kellerman | Carriage | | + | 22.9 | + | 17.6 | ET-508, LH | |
| M04965 | Y | nd | Kellerman | Carriage | | + | 22.3 | + | 18.1 | ET-508, LH | |
| M04966 | Y | nd | Kellerman | Carriage | | + | 22.1 | + | 17.5 | ET-508, LH | |
| M04967 | Y | nd | Kellerman | Carriage | | + | 22.7 | + | 18.3 | ET-836, LH | |
| M04968 | Y | nd | Kellerman | Carriage | | + | 21.4 | + | 16.9 | ET-508, LH | |
| M04969 | Y | nd | Kellerman | Carriage | | + | 22.9 | + | 18.7 | ET-508, LH | |
| M04970 | Y | nd | Kellerman | Carriage | | + | 21.5 | + | 16.5 | ET-508, LH | |
| M04971 | Y | nd | Kellerman | Carriage | | + | 22.6 | + | 17.3 | ET-508, LH | |
| M04972 | Y | nd | Kellerman | Carriage | | + | 23.3 | + | 17.8 | ET-833, LH | |
| M04973 | Y | nd | Kellerman | Carriage | | + | 23.5 | + | 19.2 | ET-508, LH | |
| M04974 | Y | nd | Kellerman | Carriage | | + | 23.2 | + | 18.3 | ET-508, LH | |
| M04975 | Y | nd | Kellerman | Carriage | | + | 22.8 | + | 17.4 | ET-508, LH | |
| M04976 | Y | nd | Kellerman | Carriage | | + | 23.9 | + | 18.3 | ET-508, LH | |
| M04977 | Y | nd | Kellerman | Carriage | | + | 22.0 | + | 16.8 | ET-508, LH | |
| M04978 | Y | nd | Kellerman | Carriage | | + | 22.5 | + | 16.9 | ET-837, LH | |
| M04979 | Y | nd | Kellerman | Carriage | | + | 23.9 | + | 17.9 | ET-508, LH | |
| M04980 | Y | nd | Kellerman | Carriage | | + | 23.5 | + | 18.3 | ET-508, LH | |
| M04981 | Y | nd | Kellerman | Carriage | | + | 24.9 | + | 21.4 | ET-508, LH | |
| M04991 | Y | nd | Kellerman | Carriage | | + | 22.0 | + | 16.4 | ET-501, LH | |
| M04992 | Y | nd | Kellerman | Carriage | | + | 20.5 | + | 17.2 | ET-839, LH | |
| M04993 | Y | nd | Kellerman | Carriage | | + | 22.7 | + | 18.1 | ET-508, LH | |
| M04994 | Y | nd | Kellerman | Carriage | | + | 22.7 | + | 19.6 | ET-508, LH | |
| M04995 | Y | nd | Kellerman | Carriage | | + | 22.9 | + | 18.8 | ET-508, LH | |
| M04996 | Y | nd | Kellerman | Carriage | | + | 20.6 | + | 18.0 | ET-508, LH | |
| M04997 | Y | nd | Kellerman | Carriage | | + | 22.1 | + | 18.3 | ET-508, LH | |
| M04998 | Y | nd | Kellerman | Carriage | | + | 21.6 | + | 18.2 | ET-508, LH | |
| M04999 | Y | nd | Kellerman | Carriage | | + | 22.0 | + | 17.8 | ET-508, LH | |
| M05000 | Y | nd | Kellerman | Carriage | | + | 20.5 | + | 17.4 | ET-508, LH | |
| M05001 | Y | nd | Kellerman | Carriage | | + | 21.3 | + | 17.8 | ET-508, LH | |
| M05002 | Y | nd | Kellerman | Carriage | | + | 21.5 | + | 18.2 | ET-508, LH | |
| M05003 | Y | nd | Kellerman | Carriage | | + | 20.7 | + | 17.2 | ET-508, LH | |
| M05004 | Y | nd | Kellerman | Carriage | | + | 20.8 | + | 17.8 | ET-508, LH | |
| M05005 | Y | nd | Kellerman | Carriage | | + | 21.1 | + | 17.4 | ET-516, LH | |
| M05022 | Y | nd | Kellerman | Carriage | | + | 18.6 | + | 16.1 | ET-842, LH | |
| M05023 | Y | nd | Kellerman | Carriage | | + | 22.8 | + | 19.5 | ET-508, LH | |
| M05024 | Y | nd | Kellerman | Carriage | | + | 20.3 | + | 16.1 | ET-518, LH | |
| M05025 | Y | nd | Kellerman | Carriage | | + | 19.4 | + | 15.9 | ET-841, LH | |
| M05026 | Y | nd | Kellerman | Carriage | | + | 19.7 | + | 16.4 | ET-843, LH | |
| M05027 | Y | nd | Kellerman | Carriage | | + | 21.5 | + | 18.8 | ET-843, LH | |
| M05028 | Y | nd | Kellerman | Carriage | | + | 21.6 | + | 18.4 | ET-843, LH | |
| M05029 | Y | nd | Kellerman | Carriage | | + | 22.0 | + | 19.1 | ET-843, LH | |
| M05062 | Y | nd | Kellerman | Carriage | | + | 20.6 | + | 15.7 | ET-508, LH | |
| M05063 | Y | nd | Kellerman | Carriage | | + | 22.1 | + | 16.4 | ET-508, LH | |
| M05064 | Y | nd | Kellerman | Carriage | | + | 21.8 | + | 15.9 | ET-508, LH | |
| M05065 | Y | nd | Kellerman | Carriage | | + | 22.6 | + | 16.6 | ET-508, LH | |
| M05066 | Y | nd | Kellerman | Carriage | | + | 23.3 | + | 17.5 | ET-508, LH | |
| M05067 | Y | nd | Kellerman | Carriage | | + | 20.9 | + | 14.9 | ET-501, LH | |
| M05068 | Y | nd | Kellerman | Carriage | | + | 21.3 | + | 15.1 | ET-518, LH | |
| M05087 | Y | nd | Kellerman | Carriage | | + | 21.6 | + | 15.4 | ET-508, LH | |
| M05088 | Y | nd | Kellerman | Carriage | | + | 20.4 | + | 15.0 | ET-508, LH | |
| M05089 | Y | nd | Kellerman | Carriage | | + | 23.5 | + | 16.0 | ET-508, LH | |
| M05090 | Y | nd | Kellerman | Carriage | | + | 21.5 | + | 15.0 | ET-845, LH | |
| M05091 | Y | nd | Kellerman | Carriage | | + | 23.8 | + | 16.6 | ET-508, LH | |
| M05092 | Y | nd | Kellerman | Carriage | | + | 22.7 | + | 16.3 | ET-508, LH | |
| M05093 | Y | nd | Kellerman | Carriage | | + | 20.8 | + | 14.5 | ET-845, LH | |
| M05094 | Y | nd | Kellerman | Carriage | | + | 22.8 | + | 16.7 | ET-822, LH | |
| M05095 | Y | nd | Kellerman | Carriage | | + | 21.7 | + | 14.9 | ET-822, LH | |
| M04923 | Z | nd | Kellerman | Carriage | | + | 20.6 | + | 18.9 | ET-832, LH | |
| M05014 | Z | nd | Kellerman | Carriage | | + | 18.3 | + | 14.7 | ET-840, LH | |
| M05032 | Z | nd | Kellerman | Carriage | | + | 20.1 | + | 16.4 | ET-844, LH | |
| M15415 | NG | NG | GA | Carriage | | - | No C_t_ | + | 16.4 | LH | |
| M15420 | NG | NG | GA | Carriage | | - | No C_t_ | + | 16.9 | LH | |
| M15421 | NG | NG | GA | Carriage | | - | No C_t_ | + | 16.9 | LH | |
| M15422 | NG | NG | GA | Carriage | | - | No C_t_ | + | 15.7 | LH | |
| M15425 | NG | NG | GA | Carriage | | - | No C_t_ | + | 15.6 | LH | |
| M15433 | NG | NG | GA | Carriage | | - | No C_t_ | + | 13.5 | LH | |
| M15436 | NG | NG | GA | Carriage | | - | No C_t_ | + | 17.6 | LH | |
| M15443 | NG | NG | GA | Carriage | | - | No C_t_ | + | 14.7 | LH | |
| M15448 | NG | NG | GA | Carriage | | - | No C_t_ | + | 15.8 | LH | |
| M15453 | NG | NG | GA | Carriage | | - | No C_t_ | - | No C_t_ | LH | |
| M15459 | NG | NG | GA | Carriage | | - | No C_t_ | + | 15.5 | LH | |
| M15460 | NG | NG | GA | Carriage | | - | No C_t_ | + | 16.0 | LH | |
| M15462 | NG | NG | GA | Carriage | | - | No C_t_ | + | 16.9 | LH | |
| M15464 | NG | NG | GA | Carriage | | - | No C_t_ | + | 16.4 | LH | |
| M15467 | NG | NG | GA | Carriage | | - | No C_t_ | + | 16.0 | LH | |
| M15647 | NG | NG | GA | Carriage | | - | No C_t_ | + | 16.5 | LH | |
| M15746 | NG | NG | GA | Carriage | | - | No C_t_ | + | 17.3 | LH | |
| M15762 | NG | NG | GA | Carriage | | - | No C_t_ | - | No C_t_ | LH | |
| M15763 | NG | NG | GA | Carriage | | - | No C_t_ | + | 18.0 | LH | |
| M15765 | NG | NG | GA | Carriage | | - | No C_t_ | + | 16.9 | LH | |
| M15767 | NG | NG | GA | Carriage | | - | No C_t_ | + | 17.9 | LH | |
| M15769 | NG | NG | GA | Carriage | | - | No C_t_ | + | 17.3 | LH | |
| M15780 | NG | NG | GA | Carriage | | - | No C_t_ | + | 15.4 | LH | |
| M15781 | NG | NG | GA | Carriage | | - | No C_t_ | + | 16.0 | LH | |
| M15812 | NG | NG | GA | Carriage | | - | No C_t_ | + | 17.5 | LH | |
| M15824 | NG | NG | GA | Carriage | | - | No C_t_ | + | 16.2 | LH | |
| M15825 | NG | NG | GA | Carriage | | - | No C_t_ | + | 16.0 | LH | |
| M15826 | NG | NG | GA | Carriage | | - | No C_t_ | + | 17.0 | LH | |
| M15827 | NG | NG | GA | Carriage | | - | No C_t_ | + | 16.7 | LH | |
| M15828 | NG | NG | GA | Carriage | | - | No C_t_ | + | 16.9 | LH | |
| M15830 | NG | NG | GA | Carriage | | - | No C_t_ | + | 17.2 | LH | |
| M16145 | NG | NG | GA | Carriage | | - | No C_t_ | + | 16.2 | LH | |
| M16146 | NG | NG | GA | Carriage | | - | No C_t_ | + | 16.8 | LH | |
| M16147 | NG | NG | GA | Carriage | | - | No C_t_ | + | 16.9 | LH | |
| M16159 | NG | NG | GA | Carriage | | - | No C_t_ | + | 17.4 | LH | |
| M16179 | NG | NG | GA | Carriage | | - | No C_t_ | + | 15.9 | LH | |
| M16219 | NG | NG | GA | Carriage | | - | No C_t_ | + | 16.5 | LH | |
| M16220 | NG | NG | GA | Carriage | | - | No C_t_ | + | 16.0 | LH | |
| M16221 | NG | NG | GA | Carriage | | - | No C_t_ | + | 16.7 | LH | |
| M16224 | NG | NG | GA | Carriage | | - | No C_t_ | + | 18.0 | LH | |
| M16227 | NG | NG | GA | Carriage | | - | No C_t_ | + | 17.3 | LH | |
| M16229 | NG | NG | GA | Carriage | | - | No C_t_ | + | 16.6 | LH | |
| M16235 | NG | NG | GA | Carriage | | - | No C_t_ | + | 17.0 | LH | |
| M16238 | NG | NG | GA | Carriage | | - | No C_t_ | + | 16.1 | LH | |
| M16268 | NG | NG | GA | Carriage | | - | No C_t_ | + | 17.6 | LH | |
| M15829 | NG | B | GA | Carriage | | - | No C_t_ | + | 16.8 | LH | |
| M15833 | NG | B | GA | Carriage | | - | No C_t_ | + | 17.8 | LH | |
| M16230 | NG | B | GA | Carriage | | - | No C_t_ | + | 18.8 | LH | |
| M16231 | NG | B | GA | Carriage | | - | No C_t_ | + | 17.3 | LH | |
| M16226 | NG | X | GA | Carriage | | - | No C_t_ | + | 17.7 | LH | |
| M15635 | NG | Y | GA | Carriage | | - | No C_t_ | + | 17.5 | LH | |
| M15636 | NG | Y | GA | Carriage | | - | No C_t_ | + | 18.1 | LH | |
| M15644 | NG | Y | GA | Carriage | | - | No C_t_ | + | 17.7 | LH | |
| M15646 | NG | Y | GA | Carriage | | - | No C_t_ | + | 17.9 | LH | |
| M16156 | NG | Y | GA | Carriage | | - | No C_t_ | + | 16.7 | LH | |
| M15416 | NG | NG | GA | Carriage | | + | 17.7 | + | 15.8 | LH | |
| M15418 | NG | NG | GA | Carriage | | + | 19.1 | + | 16.7 | LH | |
| M15426 | NG | NG | GA | Carriage | | + | 17.9 | + | 16.1 | LH | |
| M15427 | NG | NG | GA | Carriage | | + | 19.0 | + | 16.5 | LH | |
| M15428 | NG | NG | GA | Carriage | | + | 18.1 | + | 15.6 | LH | |
| M15429 | NG | NG | GA | Carriage | | + | 18.4 | + | 16.3 | LH | |
| M15435 | NG | NG | GA | Carriage | | + | 18.2 | + | 15.6 | LH | |
| M15438 | NG | NG | GA | Carriage | | + | 18.1 | + | 16.6 | LH | |
| M15440 | NG | NG | GA | Carriage | | + | 16.4 | + | 16.0 | LH | |
| M15444 | NG | NG | GA | Carriage | | + | 17.8 | + | 15.4 | LH | |
| M15449 | NG | NG | GA | Carriage | | + | 18.7 | + | 16.7 | LH | |
| M15457 | NG | NG | GA | Carriage | | + | 16.6 | + | 15.2 | LH | |
| M15461 | NG | NG | GA | Carriage | | + | 17.6 | + | 17.1 | LH | |
| M15465 | NG | NG | GA | Carriage | | + | 17.2 | + | 16.2 | LH | |
| M15471 | NG | NG | GA | Carriage | | + | 17.8 | + | 17.2 | LH | |
| M15640 | NG | NG | GA | Carriage | | + | 18.6 | + | 17.4 | LH | |
| M15645 | NG | NG | GA | Carriage | | + | 19.4 | + | 16.8 | LH | |
| M15649 | NG | NG | GA | Carriage | | + | 18.4 | + | 17.0 | LH | |
| M15761 | NG | NG | GA | Carriage | | + | 18.0 | + | 18.2 | LH | |
| M15777 | NG | NG | GA | Carriage | | + | 17.5 | + | 18.1 | LH | |
| M15788 | NG | NG | GA | Carriage | | + | 18.0 | + | 17.4 | LH | |
| M15832 | NG | NG | GA | Carriage | | + | 16.7 | + | 16.4 | LH | |
| M15836 | NG | NG | GA | Carriage | | + | 16.9 | + | 17.3 | LH | |
| M15838 | NG | NG | GA | Carriage | | + | 17.4 | + | 16.9 | LH | |
| M16148 | NG | NG | GA | Carriage | | + | 18.3 | + | 16.7 | LH | |
| M16162 | NG | NG | GA | Carriage | | + | 15.5 | + | 16.6 | LH | |
| M16163 | NG | NG | GA | Carriage | | + | 16.4 | + | 17.1 | LH | |
| M16165 | NG | NG | GA | Carriage | | + | 15.2 | + | 16.3 | LH | |
| M16175 | NG | NG | GA | Carriage | | + | 16.5 | + | 17.0 | LH | |
| M16225 | NG | NG | GA | Carriage | | + | 14.7 | + | 17.5 | LH | |
| M16233 | NG | NG | GA | Carriage | | + | 34.0 | + | 15.6 | LH | |
| M16237 | NG | NG | GA | Carriage | | + | 13.5 | + | 16.8 | LH | |
| M16239 | NG | NG | GA | Carriage | | + | 18.0 | + | 17.3 | LH | |
| M16240 | NG | NG | GA | Carriage | | + | 19.0 | + | 17.3 | LH | |
| M16241 | NG | NG | GA | Carriage | | + | 14.5 | + | 17.1 | LH | |
| M16242 | NG | NG | GA | Carriage | | + | 19.0 | + | 18.3 | LH | |
| M16267 | NG | NG | GA | Carriage | | + | 17.0 | + | 17.5 | LH | |
| M15423 | NG | B | GA | Carriage | | + | 19.3 | + | 16.5 | LH | |
| M15424 | NG | B | GA | Carriage | | + | 19.5 | + | 17.3 | LH | |
| M15439 | NG | B | GA | Carriage | | + | 17.4 | + | 16.3 | LH | |
| M15445 | NG | B | GA | Carriage | | + | 19.2 | + | 17.1 | LH | |
| M15446 | NG | B | GA | Carriage | | + | 18.0 | + | 16.0 | LH | |
| M15450 | NG | B | GA | Carriage | | + | 19.7 | + | 16.3 | LH | |
| M15454 | NG | B | GA | Carriage | | + | 17.8 | + | 16.3 | LH | |
| M15455 | NG | B | GA | Carriage | | + | 16.8 | + | 16.3 | LH | |
| M15456 | NG | B | GA | Carriage | | + | 17.9 | + | 16.6 | LH | |
| M15468 | NG | B | GA | Carriage | | + | 16.7 | + | 16.0 | LH | |
| M15638 | NG | B | GA | Carriage | | + | 18.8 | + | 18.1 | LH | |
| M15748 | NG | B | GA | Carriage | | + | 17.4 | + | 16.9 | LH | |
| M15764 | NG | B | GA | Carriage | | + | 17.8 | + | 17.6 | LH | |
| M15766 | NG | B | GA | Carriage | | + | 18.8 | + | 17.2 | LH | |
| M15835 | NG | B | GA | Carriage | | + | 18.9 | + | 19.0 | LH | |
| M16149 | NG | B | GA | Carriage | | + | 18.5 | + | 17.4 | LH | |
| M16178 | NG | B | GA | Carriage | | + | 16.7 | + | 17.9 | LH | |
| M16223 | NG | B | GA | Carriage | | + | 14.3 | + | 17.4 | LH | |
| M16272 | NG | B | GA | Carriage | | + | 20.0 | + | 20.3 | LH | |
| M15785 | NG | C | GA | Carriage | | + | 16.7 | + | 17.0 | LH | |
| M15473 | NG | Y | GA | Carriage | | + | 17.1 | + | 17.3 | LH | |
| M15637 | NG | Y | GA | Carriage | | + | 16.6 | + | 17.5 | LH | |
| M15639 | NG | Y | GA | Carriage | | + | 17.3 | + | 17.0 | LH | |
| M15834 | NG | Y | GA | Carriage | | + | 18.3 | + | 17.9 | LH | |
| M16161 | NG | Y | GA | Carriage | | + | 15.7 | + | 15.8 | LH | |
| M16176  M16164 | NG  NG | Y  Y | GA  GA | Carriage  Carriage | | +  + | 17.3  16.4 | +  + | 17.3  15.6 | LH  LH | |
| M16177 | NG | Y | GA | Carriage | | + | 16.2 | + | 16.6 | LH | |
| M16236 | NG | Y | GA | Carriage | | + | 19.0 | + | 18.1 | LH | |
| M15417 | B | B | GA | Carriage | | + | 15.8 | + | 16.1 | LH | |
| M15430 | B | B | GA | Carriage | | + | 16.7 | + | 15.6 | LH | |
| M15431 | B | B | GA | Carriage | | + | 16.4 | + | 15.5 | LH | |
| M15432 | B | B | GA | Carriage | | + | 17.2 | + | 15.6 | LH | |
| M15447 | B | B | GA | Carriage | | + | 17.3 | + | 15.2 | LH | |
| M15451 | B | B | GA | Carriage | | + | 17.4 | + | 14.7 | LH | |
| M15463 | B | B | GA | Carriage | | + | 17.0 | + | 16.0 | LH | |
| M15466 | B | B | GA | Carriage | | + | 16.7 | + | 15.5 | LH | |
| M15472 | B | B | GA | Carriage | | + | 17.3 | + | 16.2 | LH | |
| M15831 | B | B | GA | Carriage | | + | 16.3 | + | 15.1 | LH | |
| M15837 | B | B | GA | Carriage | | + | 16.9 | + | 16.0 | LH | |
| M16157 | B | B | GA | Carriage | | + | 14.7 | + | 16.1 | LH | |
| M16158 | B | B | GA | Carriage | | + | 17.3 | + | 17.4 | LH | |
| M16228 | B | B | GA | Carriage | | + | 14.7 | + | 15.6 | LH | |
| M15437 | Y | Y | GA | Carriage | | + | 18.4 | + | 16.7 | LH | |
| M15641 | Y | Y | GA | Carriage | | + | 16.5 | + | 17.2 | LH | |
| M15650 | Y | Y | GA | Carriage | | + | 19.1 | + | 18.7 | LH | |
| M15782 | Y | Y | GA | Carriage | | + | 17.5 | + | 18.4 | LH | |
| M16174 | Y | Y | GA | Carriage | | + | 18.4 | + | 17.7 | LH | |
| M16234 | Y | Y | GA | Carriage | | + | 16.8 | + | 17.4 | LH | |
| M16160 | 29E | n/a^8^ | GA | Carriage | | + | 14.2 | + | 16.6 | LH | |
| M17258 | NG | NG | MD | Carriage | | - | No C_t_ | + | 17.0 | LH | |
| M17262 | NG | NG | MD | Carriage | | - | No C_t_ | + | 20.0 | LH | |
| M17264 | NG | NG | MD | Carriage | | - | No C_t_ | + | 20.1 | LH | |
| M17270 | NG | NG | MD | Carriage | | - | No C_t_ | + | 17.5 | LH | |
| M17271 | NG | NG | MD | Carriage | | - | No C_t_ | + | 18.4 | LH | |
| M17277 | NG | NG | MD | Carriage | | - | No C_t_ | + | 15.5 | LH | |
| M17281 | NG | NG | MD | Carriage | | - | No C_t_ | + | 16.5 | LH | |
| M17285 | NG | NG | MD | Carriage | | - | No C_t_ | + | 17.1 | LH | |
| M17286 | NG | NG | MD | Carriage | | - | No C_t_ | + | 18.0 | LH | |
| M17288 | NG | NG | MD | Carriage | | - | No C_t_ | + | 16.1 | LH | |
| M17289 | NG | NG | MD | Carriage | | - | No C_t_ | + | 16.8 | LH | |
| M17290 | NG | NG | MD | Carriage | | - | No C_t_ | + | 17.2 | LH | |
| M17292 | NG | NG | MD | Carriage | | - | No C_t_ | + | 17.3 | LH | |
| M17293 | NG | NG | MD | Carriage | | - | No C_t_ | + | 15.5 | LH | |
| M17296 | NG | NG | MD | Carriage | | - | No C_t_ | + | 17.5 | LH | |
| M17303 | NG | NG | MD | Carriage | | - | No C_t_ | + | 16.5 | LH | |
| M17305 | NG | NG | MD | Carriage | | - | No C_t_ | + | 17.4 | LH | |
| M17308 | NG | NG | MD | Carriage | | - | No C_t_ | + | 16.7 | LH | |
| M17309 | NG | NG | MD | Carriage | | - | No C_t_ | + | 16.1 | LH | |
| M17310 | NG | NG | MD | Carriage | | - | No C_t_ | + | 17.4 | LH | |
| M17313 | NG | NG | MD | Carriage | | - | No C_t_ | + | 17.7 | LH | |
| M17321 | NG | NG | MD | Carriage | | - | No C_t_ | + | 15.8 | LH | |
| M17324 | NG | NG | MD | Carriage | | - | No C_t_ | + | 18.1 | LH | |
| M17326 | NG | NG | MD | Carriage | | - | No C_t_ | + | 17.0 | LH | |
| M17328 | NG | NG | MD | Carriage | | - | No C_t_ | + | 17.8 | LH | |
| M17331 | NG | NG | MD | Carriage | | - | No C_t_ | + | 16.3 | LH | |
| M17335 | NG | NG | MD | Carriage | | - | No C_t_ | + | 20.5 | LH | |
| M17337 | NG | NG | MD | Carriage | | - | No C_t_ | + | 17.5 | LH | |
| M17339 | NG | NG | MD | Carriage | | - | No C_t_ | + | 18.9 | LH | |
| M17340 | NG | NG | MD | Carriage | | - | No C_t_ | + | 18.0 | LH | |
| M17343 | NG | NG | MD | Carriage | | - | No C_t_ | + | 20.4 | LH | |
| M17344 | NG | NG | MD | Carriage | | - | No C_t_ | + | 16.8 | LH | |
| M17345 | NG | NG | MD | Carriage | | - | No C_t_ | + | 18.1 | LH | |
| M17347 | NG | NG | MD | Carriage | | - | No C_t_ | + | 18.0 | LH | |
| M17348 | NG | NG | MD | Carriage | | - | No C_t_ | + | 21.2 | LH | |
| M17349 | NG | NG | MD | Carriage | | - | No C_t_ | + | 17.1 | LH | |
| M17350 | NG | NG | MD | Carriage | | - | No C_t_ | + | 17.4 | LH | |
| M17351 | NG | NG | MD | Carriage | | - | No C_t_ | + | 17.9 | LH | |
| M17353 | NG | NG | MD | Carriage | | - | No C_t_ | + | 18.7 | LH | |
| M17354 | NG | NG | MD | Carriage | | - | No C_t_ | + | 19.6 | LH | |
| M17355 | NG | NG | MD | Carriage | | - | No C_t_ | + | 18.4 | LH | |
| M17361 | NG | NG | MD | Carriage | | - | No C_t_ | + | 18.9 | LH | |
| M17362 | NG | NG | MD | Carriage | | - | No C_t_ | + | 15.8 | LH | |
| M17364 | NG | NG | MD | Carriage | | - | No C_t_ | + | 18.9 | LH | |
| M17365 | NG | NG | MD | Carriage | | - | No C_t_ | + | 16.8 | LH | |
| M17367 | NG | NG | MD | Carriage | | - | No C_t_ | + | 16.0 | LH | |
| M17370 | NG | NG | MD | Carriage | | - | No C_t_ | + | 16.2 | LH | |
| M17381 | NG | NG | MD | Carriage | | - | No C_t_ | + | 15.3 | LH | |
| M17385 | NG | NG | MD | Carriage | | - | No C_t_ | + | 16.4 | LH | |
| M17395 | NG | NG | MD | Carriage | | - | No C_t_ | + | 15.6 | LH | |
| M17399 | NG | NG | MD | Carriage | | - | No C_t_ | + | 18.4 | LH | |
| M17401 | NG | NG | MD | Carriage | | - | No C_t_ | + | 16.4 | LH | |
| M17403 | NG | NG | MD | Carriage | | - | No C_t_ | + | 16.7 | LH | |
| M17404 | NG | NG | MD | Carriage | | - | No C_t_ | + | 14.6 | LH | |
| M17405 | NG | NG | MD | Carriage | | - | No C_t_ | + | 17.4 | LH | |
| M17406 | NG | NG | MD | Carriage | | - | No C_t_ | + | 15.5 | LH | |
| M17408 | NG | NG | MD | Carriage | | - | No C_t_ | + | 15.3 | LH | |
| M17411 | NG | NG | MD | Carriage | | - | No C_t_ | + | 15.6 | LH | |
| M17412 | NG | NG | MD | Carriage | | - | No C_t_ | + | 14.9 | LH | |
| M17414 | NG | NG | MD | Carriage | | - | No C_t_ | + | 15.5 | LH | |
| M17415 | NG | NG | MD | Carriage | | - | No C_t_ | + | 16.7 | LH | |
| M17417 | NG | NG | MD | Carriage | | - | No C_t_ | + | 18.3 | LH | |
| M17418 | NG | NG | MD | Carriage | | - | No C_t_ | + | 16.1 | LH | |
| M17419 | NG | NG | MD | Carriage | | - | No C_t_ | + | 15.6 | LH | |
| M17425 | NG | NG | MD | Carriage | | - | No C_t_ | + | 14.8 | LH | |
| M17426 | NG | NG | MD | Carriage | | - | No C_t_ | + | 16.8 | LH | |
| M17427 | NG | NG | MD | Carriage | | - | No C_t_ | + | 15.6 | LH | |
| M17437 | NG | NG | MD | Carriage | | - | No C_t_ | + | 16.2 | LH | |
| M17439 | NG | NG | MD | Carriage | | - | No C_t_ | + | 13.9 | LH | |
| M17442 | NG | NG | MD | Carriage | | - | No C_t_ | + | 14.4 | LH | |
| M17443 | NG | NG | MD | Carriage | | - | No C_t_ | + | 15.2 | LH | |
| M17433 | NG | Y | MD | Carriage | | - | No C_t_ | + | 17.2 | LH | |
| M17257 | NG | NG | MD | Carriage | | + | 18.1 | + | 19.0 | LH | |
| M17259 | NG | NG | MD | Carriage | | + | 18.5 | + | 18.8 | LH | |
| M17273 | NG | NG | MD | Carriage | | + | 19.4 | + | 16.9 | LH | |
| M17274 | NG | NG | MD | Carriage | | + | 17.2 | + | 18.0 | LH | |
| M17298 | NG | NG | MD | Carriage | | + | 15.0 | + | 16.6 | LH | |
| M17302 | NG | NG | MD | Carriage | | + | 18.2 | + | 16.4 | LH | |
| M17317 | NG | NG | MD | Carriage | | + | 16.0 | + | 17.6 | LH | |
| M17318 | NG | NG | MD | Carriage | | + | 18.3 | + | 17.1 | LH | |
| M17320 | NG | NG | MD | Carriage | | + | 17.0 | + | 17.0 | LH | |
| M17322 | NG | NG | MD | Carriage | | + | 15.5 | + | 16.7 | LH | |
| M17330 | NG | NG | MD | Carriage | | + | 16.5 | + | 18.7 | LH | |
| M17357 | NG | NG | MD | Carriage | | + | 16.2 | + | 17.7 | LH | |
| M17363 | NG | NG | MD | Carriage | | + | 15.5 | + | 18.3 | LH | |
| M17378 | NG | NG | MD | Carriage | | + | 15.4 | + | 16.9 | LH | |
| M17380 | NG | NG | MD | Carriage | | + | 15.8 | + | 16.5 | LH | |
| M17386 | NG | NG | MD | Carriage | | + | 15.5 | + | 15.8 | LH | |
| M17388 | NG | NG | MD | Carriage | | + | 15.8 | + | 17.6 | LH | |
| M17390 | NG | NG | MD | Carriage | | + | 15.7 | + | 17.2 | LH | |
| M17391 | NG | NG | MD | Carriage | | + | 15.8 | + | 15.6 | LH | |
| M17392 | NG | NG | MD | Carriage | | + | 17.8 | + | 19.6 | LH | |
| M17394 | NG | NG | MD | Carriage | | + | 17.7 | + | 15.0 | LH | |
| M17396 | NG | NG | MD | Carriage | | + | 16.0 | + | 16.1 | LH | |
| M17410 | NG | NG | MD | Carriage | | + | 15.4 | + | 17.6 | LH | |
| M17416 | NG | NG | MD | Carriage | | + | 16.2 | + | 15.0 | LH | |
| M17423 | NG | NG | MD | Carriage | | + | 15.8 | + | 16.8 | LH | |
| M17428 | NG | NG | MD | Carriage | | + | 15.4 | + | 14.8 | LH | |
| M17429 | NG | NG | MD | Carriage | | + | 16.3 | + | 16.3 | LH | |
| M17430 | NG | NG | MD | Carriage | | + | 16.3 | + | 17.1 | LH | |
| M17436 | NG | NG | MD | Carriage | | + | 15.7 | + | 14.9 | LH | |
| M17256 | NG | B | MD | Carriage | | + | 17.1 | + | 19.1 | LH | |
| M17261 | NG | B | MD | Carriage | | + | 21.5 | + | 18.9 | LH | |
| M17263 | NG | B | MD | Carriage | | + | 18.6 | + | 19.0 | LH | |
| M17268 | NG | B | MD | Carriage | | + | 16.2 | + | 17.5 | LH | |
| M17272 | NG | B | MD | Carriage | | + | 17.8 | + | 18.7 | LH | |
| M17278 | NG | B | MD | Carriage | | + | 22.1 | + | 17.9 | LH | |
| M17284 | NG | B | MD | Carriage | | + | 16.4 | + | 17.4 | LH | |
| M17291 | NG | B | MD | Carriage | | + | 18.5 | + | 17.0 | LH | |
| M17295 | NG | B | MD | Carriage | | + | 19.0 | + | 16.7 | LH | |
| M17297 | NG | B | MD | Carriage | | + | 17.0 | + | 17.3 | LH | |
| M17300 | NG | B | MD | Carriage | | + | 16.2 | + | 16.5 | LH | |
| M17301 | NG | B | MD | Carriage | | + | 20.8 | + | 18.0 | LH | |
| M17306 | NG | B | MD | Carriage | | + | 16.3 | + | 18.4 | LH | |
| M17311 | NG | B | MD | Carriage | | + | 17.4 | + | 17.5 | LH | |
| M17312 | NG | B | MD | Carriage | | + | 16.8 | + | 17.1 | LH | |
| M17316 | NG | B | MD | Carriage | | + | 15.3 | + | 18.1 | LH | |
| M17325 | NG | B | MD | Carriage | | + | 17.8 | + | 17.4 | LH | |
| M17336 | NG | B | MD | Carriage | | + | 24.4 | + | 17.4 | LH | |
| M17346 | NG | B | MD | Carriage | | + | 15.7 | + | 17.7 | LH | |
| M17356 | NG | B | MD | Carriage | | + | 18.0 | + | 17.4 | LH | |
| M17358 | NG | B | MD | Carriage | | + | 16.0 | + | 18.7 | LH | |
| M17360 | NG | B | MD | Carriage | | + | 16.1 | + | 15.3 | LH | |
| M17366 | NG | B | MD | Carriage | | + | 17.0 | + | 16.0 | LH | |
| M17368 | NG | B | MD | Carriage | | + | 16.9 | + | 16.5 | LH | |
| M17371 | NG | B | MD | Carriage | | + | 16.0 | + | 17.6 | LH | |
| M17374 | NG | B | MD | Carriage | | + | 17.0 | + | 15.7 | LH | |
| M17377 | NG | B | MD | Carriage | | + | 16.7 | + | 16.6 | LH | |
| M17383 | NG | B | MD | Carriage | | + | 15.5 | + | 18.4 | LH | |
| M17400 | NG | B | MD | Carriage | | + | 19.3 | + | 16.7 | LH | |
| M17407 | NG | B | MD | Carriage | | + | 15.5 | + | 15.6 | LH | |
| M17409 | NG | B | MD | Carriage | | + | 15.8 | + | 16.2 | LH | |
| M17420 | NG | B | MD | Carriage | | + | 26.5 | + | 15.2 | LH | |
| M17424 | NG | B | MD | Carriage | | + | 15.8 | + | 16.0 | LH | |
| M17432 | NG | B | MD | Carriage | | + | 16.6 | + | 16.3 | LH | |
| M17434 | NG | B | MD | Carriage | | + | 16.1 | + | 16.1 | LH | |
| M17435 | NG | B | MD | Carriage | | + | 15.5 | + | 15.3 | LH | |
| M17334 | NG | C | MD | Carriage | | + | 19.3 | + | 18.2 | LH | |
| M17372 | NG | C | MD | Carriage | | + | 15.9 | + | 17.6 | LH | |
| M17393 | NG | C | MD | Carriage | | + | 15.6 | + | 17.8 | LH | |
| M17265 | NG | Y | MD | Carriage | | + | 22.4 | + | 19.1 | LH | |
| M17269 | NG | Y | MD | Carriage | | + | 19.6 | + | 18.2 | LH | |
| M17280 | NG | Y | MD | Carriage | | + | 21.1 | + | 16.2 | LH | |
| M17282 | NG | Y | MD | Carriage | | + | 23.4 | + | 17.1 | LH | |
| M17287 | NG | Y | MD | Carriage | | + | 19.2 | + | 16.9 | LH | |
| M17299 | NG | Y | MD | Carriage | | + | 20.8 | + | 20.7 | LH | |
| M17314 | NG | Y | MD | Carriage | | + | 19.3 | + | 20.1 | LH | |
| M17315 | NG | Y | MD | Carriage | | + | 17.5 | + | 17.3 | LH | |
| M17332 | NG | Y | MD | Carriage | | + | 17.7 | + | 17.4 | LH | |
| M17338 | NG | Y | MD | Carriage | | + | 18.4 | + | 16.8 | LH | |
| M17341 | NG | Y | MD | Carriage | | + | 16.4 | + | 18.8 | LH | |
| M17342 | NG | Y | MD | Carriage | | + | 16.4 | + | 18.1 | LH | |
| M17352 | NG | Y | MD | Carriage | | + | 17.2 | + | 18.5 | LH | |
| M17359 | NG | Y | MD | Carriage | | + | 16.9 | + | 19.1 | LH | |
| M17373 | NG | Y | MD | Carriage | | + | 15.9 | + | 17.5 | LH | |
| M17375 | NG | Y | MD | Carriage | | + | 16.8 | + | 16.0 | LH | |
| M17376 | NG | Y | MD | Carriage | | + | 16.3 | + | 15.8 | LH | |
| M17379 | NG | Y | MD | Carriage | | + | 16.8 | + | 15.8 | LH | |
| M17382 | NG | Y | MD | Carriage | | + | 17.3 | + | 15.4 | LH | |
| M17387 | NG | Y | MD | Carriage | | + | 16.3 | + | 17.9 | LH | |
| M17397 | NG | Y | MD | Carriage | | + | 15.6 | + | 18.2 | LH | |
| M17413 | NG | Y | MD | Carriage | | + | 16.1 | + | 16.2 | LH | |
| M17421 | NG | Y | MD | Carriage | | + | 17.3 | + | 17.1 | LH | |
| M17422 | NG | Y | MD | Carriage | | + | 18.9 | + | 16.4 | LH | |
| M17438 | NG | Y | MD | Carriage | | + | 16.3 | + | 15.9 | LH | |
| M17266 | Y | Y | MD | Carriage | | + | 22.4 | + | 19.3 | LH | |
| M17275 | Y | Y | MD | Carriage | | + | 17.2 | + | 19.1 | LH | |
| M17276 | Y | Y | MD | Carriage | | + | 21.9 | + | 18.1 | LH | |
| M17283 | Y | Y | MD | Carriage | | + | 19.8 | + | 17.6 | LH | |
| M17294 | Y | Y | MD | Carriage | | + | 21.7 | + | 16.2 | LH | |
| M17304 | Y | Y | MD | Carriage | | + | 18.7 | + | 16.8 | LH | |
| M17307 | Y | Y | MD | Carriage | | + | 18.2 | + | 16.2 | LH | |
| M17319 | Y | Y | MD | Carriage | | + | 17.3 | + | 17.5 | LH | |
| M17323 | Y | Y | MD | Carriage | | + | 18.1 | + | 17.9 | LH | |
| M17327 | Y | Y | MD | Carriage | | + | 19.3 | + | 19.6 | LH | |
| M17329 | Y | Y | MD | Carriage | | + | 16.2 | + | 17.5 | LH | |
| M17333 | Y | Y | MD | Carriage | | + | 17.1 | + | 21.6 | LH | |
| M17369 | Y | Y | MD | Carriage | | + | 15.8 | + | 16.4 | LH | |
| M17384 | Y | Y | MD | Carriage | | + | 15.8 | + | 16.6 | LH | |
| M17389 | Y | Y | MD | Carriage | | + | 18.2 | + | 16.7 | LH | |
| M17398 | Y | Y | MD | Carriage | | + | 15.5 | + | 18.2 | LH | |
| M17402 | Y | Y | MD | Carriage | | + | 19.5 | + | 16.2 | LH | |
| M17431 | Y | Y | MD | Carriage | | + | 15.1 | + | 18.8 | LH | |
| M17441 | Y | Y | MD | Carriage | | + | 15.9 | + | 15.0 | LH | |
| M11882 | NG | NG | FL | Invasive^9^ | | - | No C_t_ | + | 18.7 | MLST | |
| M11997 | NG | NG | PA | Non-invasive^10^ | | - | No C_t_ | + | 17.2 | MLST | |
| M12067 | NG | NG | TN | Non-invasive | | - | No Ct | + | 18.2 | MLST | |
| M12143 | NG | NG | TN | Invasive | | - | No Ct | + | 18.5 | MLST | |
| M12285 | NG | NG | MD | Invasive | | - | No C_t_ | + | 17.9 | MLST | |
| M13600 | NG | NG | UT | Non-invasive | | - | No Ct | + | 20.6 | MLST | |
| M13860 | NG | NG | UT | Non-invasive | | - | No Ct | + | 21.1 | MLST | |
| M13876 | NG | NG | UT | Non-invasive | | - | No Ct | + | 23.0 | MLST | |
| M13888 | NG | NG | UT | Non-invasive | | - | No C_t_ | + | 21.6 | MLST | |
| M13889 | NG | NG | UT | Non-invasive | | - | No C_t_ | + | 20.0 | MLST | |
| M13893 | NG | NG | UT | Non-invasive | | - | No C_t_ | + | 19.8 | MLST | |
| M13895 | NG | NG | UT | Non-invasive | | - | No C_t_ | + | 20.1 | MLST | |
| M13976 | NG | NG | Brazil | Invasive | | - | No Ct | + | 22.1 | MLST | |
| M13979 | NG | NG | Brazil | Invasive | | - | No Ct | + | 22.1 | MLST | |
| M13992 | NG | NG | Brazil | Invasive | | - | No Ct | + | 21.8 | MLST | |
| M14726 | NG | NG | OH | Invasive | | - | No C_t_ | + | 22.8 | MLST | |
| M15040 | NG | NG | GA/ABCs^11^ | Invasive | | - | No C_t_ | + | 19.1 | MLST | |
| M15054 | NG | NG | South Africa | Invasive | | - | No C_t_ | + | 19.5 | MLST | |
| M15114 | NG | NG | NY | Non-invasive | | - | No C_t_ | + | 19.4 | MLST | |
| M15116 | NG | NG | NY | Non-invasive | | - | No C_t_ | + | 21.0 | MLST | |
| M15117 | NG | NG | NY | Non-invasive | | - | No C_t_ | + | 19.7 | MLST | |
| M15122 | NG | NG | NY | Non-invasive | | - | No C_t_ | + | 18.7 | MLST | |
| M15123 | NG | NG | NY | Non-invasive | | - | No C_t_ | + | 20.1 | MLST | |
| M15124 | NG | NG | NY | Non-invasive | | - | No C_t_ | + | 18.8 | MLST | |
| M15126 | NG | NG | NY | Non-invasive | | - | No C_t_ | + | 18.9 | MLST | |
| M15130 | NG | NG | NY | Non-invasive | | - | No C_t_ | + | 20.6 | MLST | |
| M06854 | NG | Y | GA/ABCs | Invasive | | + | 20.0 | + | 19.4 | None | |
| M07060 | A | A | NY | Invasive | | + | 18.3 | + | 18.1 | None | |
| M07221 | A | A | MI | Non-invasive | | + | 17.7 | + | 18.4 | None | |
| M07542 | A | A | NY | Invasive | | + | 13.8 | + | 13.0 | None | |
| M07796 | A | A | Cameroon | Invasive | | + | 19.5 | + | 21.9 | None | |
| M10055 | A | A | Bangladesh | Invasive | | + | 18.0 | + | 20.6 | None | |
| M12753 | A | A | Mali | Invasive | | + | 16.8 | + | 19.7 | None | |
| M13070 | A | A | Philippines | Invasive | | + | 16.7 | + | 20.6 | None | |
| M13156 | A | A | Bangladesh | Invasive | | + | 15.6 | + | 17.8 | None | |
| M13221 | A | A | Philippines | Invasive | | + | 19.1 | + | 19.7 | None | |
| M14418 | A | A | CT/ABCs | Invasive | | + | 18.9 | + | 19.2 | MLST | |
| M15052 | A | A | South Africa | Invasive | | + | 18.2 | + | 20.4 | None | |
| M05178 | B | B | OR/ABCs | Invasive | | + | 19.1 | + | 18.8 | serotyped, serosubtyped | |
| M07268 | B | B | GA/ABCs | Invasive | | + | 15.8 | + | 22.2 | MLST | |
| M07336 | B | B | OR/ABCs | Invasive | | + | 21.2 | + | 21.4 | MLST | |
| M07500 | B | B | OR/ABCs | Invasive | | + | 17.3 | + | 21.1 | MLST | |
| M07622 | B | B | CA/ABCs | Invasive | | + | 16.7 | + | 21.1 | MLST | |
| M08238 | B | B | Hong Kong | Invasive | | + | 18.6 | + | 23.0 | None | |
| M08579 | B | B | Croatia | Invasive | | + | 16.6 | + | 17.3 | None | |
| M08861 | B | B | NE | Invasive | | + | 16.3 | + | 20.6 | None | |
| M10928 | B | B | SC | Invasive | | + | 16.9 | + | 18.3 | None | |
| M16378 | B | B | FL | Invasive | | + | 19.5 | + | 21.0 | None | |
| M03045 | C | C | TX | Invasive | | + | 17.0 | + | 23.6 | 16S (13) | |
| M08323 | C | C | OH | Invasive | | + | 22.4 | + | 24.5 | None | |
| M08762 | C | C | GA/ABCs | Invasive | | + | 17.5 | + | 18.0 | MLST | |
| M10137 | C | C | MN/ABCs | Invasive | | + | 19.0 | + | 21.2 | MLST | |
| M10138 | C | C | MN/ABCs | Invasive | | + | 19.0 | + | 21.0 | MLST | |
| M10379 | C | C | GA/ABCs | Invasive | | + | 15.4 | + | 18.7 | MLST | |
| M10399 | C | C | MS | Invasive | | + | 16.4 | + | 18.0 | None | |
| M10861 | C | C | Brazil | Invasive | | + | 17.1 | + | 18.1 | None | |
| M10863 | C | C | Brazil | Invasive | | + | 17.8 | + | 18.2 | None | |
| M11187 | C | C | MN/ABCs | Invasive | | + | 14.9 | + | 16.6 | MLST | |
| M06049 | W135 | W135 | NE | Invasive | | + | 18.5 | + | 19.7 | None | |
| M07034 | W135 | W135 | NY | Invasive | | + | 19.9 | + | 19.9 | None | |
| M07168 | W135 | W135 | The Gambia | Invasive | | + | 15.8 | + | 16.9 | None | |
| M07999 | W135 | W135 | Burkina Faso | Invasive | | + | 20.6 | + | 21.1 | None | |
| M08236 | W135 | W135 | Hong Kong | Invasive | | + | 24.7 | + | 25.4 | None | |
| M09470 | W135 | W135 | Burkina Faso | Non-invasive | | + | 24.5 | + | 26.7 | None | |
| M09491 | W135 | W135 | Burkina Faso | Non-invasive | | + | 26.5 | + | 25.0 | None | |
| M09494 | W135 | W135 | Burkina Faso | Non-invasive | | + | 25.9 | + | 26.0 | None | |
| M12649 | W135 | W135 | MN/ABCs | Invasive | | + | 22.2 | + | 20.3 | MLST | |
| M07149 | W135 | NG | Saudi Arabia | Invasive | | + | 21.2 | + | 23.1 | None | |
| M07575 | X | X | MD/ABCs | Invasive | | + | 16.5 | + | 23.4 | MLST | |
| M08210 | X | X | NC | Invasive | | + | 18.1 | + | 19.3 | None | |
| M09554 | X | X | Burkina Faso | Non-invasive | | + | 21.5 | + | 27.2 | None | |
| M09586 | X | X | Burkina Faso | Non-invasive | | + | 25.2 | + | 29.5 | None | |
| M09590 | X | X | Burkina Faso | Non-invasive | | + | 29.7 | + | 26.6 | None | |
| M09601 | X | X | Burkina Faso | Non-invasive | | + | 21.1 | + | 25.7 | None | |
| M09621 | X | X | Burkina Faso | Non-invasive | | + | 18.0 | + | 26.6 | None | |
| M09645 | X | X | Burkina Faso | Non-invasive | | + | 16.2 | + | 26.4 | None | |
| M09664 | X | X | MN/ABCs | Invasive | | + | 17.6 | + | 20.4 | MLST | |
| M06107 | Y | Y | CA/ABCs | Invasive | | + | 12.1 | + | 17.7 | MLST | |
| M06159 | Y | Y | GA/ABCs | Invasive | | + | 15.2 | + | 19.6 | MLST | |
| M07354 | Y | Y | CT/ABCs | Invasive | | + | 18.5 | + | 20.0 | MLST | |
| M07388 | Y | Y | MD/ABCs | Invasive | | + | 17.3 | + | 20.6 | MLST | |
| M08488 | Y | Y | NY/ABCs | Invasive | | + | 17.9 | + | 24.0 | MLST | |
| M09977 | Y | Y | TN/ABCs | Invasive | | + | 13.8 | + | 20.3 | MLST | |
| M10493 | Y | Y | TN/ABCs | Invasive | | + | 16.3 | + | 20.6 | MLST | |
| M12357 | Y | Y | NM/ABCs | Invasive | | + | 15.8 | + | 19.7 | MLST | |
| M12881 | Y | Y | HI | Invasive | | + | 15.7 | + | 20.1 | None | |
| M13118 | Y | Y | NM/ABCs | Invasive | | + | 18.6 | + | 20.2 | MLST | |
| M16430 | Y | Y | CO/ABCs | Invasive | | + | 19.6 | + | 22.6 | MLST | |
| M01148 | Z | n/a^12^ | CO | Invasive | | + | 17.1 | + | 18.1 | None | |
| M03470 | Z | n/a | MD | Invasive | | + | 15.8 | + | 18.1 | serosubtyped, 16S (17) | |
| M03684 | Z | n/a | NE | Invasive | | + | 17.0 | + | 19.0 | None | |
| M03806 | Z | n/a | FL | Invasive | | + | 15.1 | + | 19.0 | None | |
| M04040 | Z | n/a | Canada | Invasive | | + | 18.0 | + | 19.1 | None | |
| M04164 | Z | n/a | GA/ABCs | Invasive | | + | 17.1 | + | 19.4 | serosubtyped | |
| M05273 | Z | n/a | GA/ABCs | Invasive | | + | 18.4 | + | 19.8 | serosubtyped | |
| M05955 | Z | n/a | MN/ABCs | Invasive | | + | 13.6 | + | 18.4 | None | |
| M15470 | Z | n/a | CO/ABCs | Invasive | | + | 19.0 | + | 21.5 | MLST | |
| M00330 | 29E | n/a | 1966^13^ | Invasive | | + | 18.3 | + | 19.4 | 16S (136) | |
| M01489 | 29E | n/a | WA | Non-invasive | | + | 17.0 | + | 18.9 | None | |
| M01490 | 29E | n/a | WA | Non-invasive | | + | 19.1 | + | 19.3 | None | |
| M01491 | 29E | n/a | WA | Non-invasive | | + | 18.2 | + | 19.9 | None | |
| M03447 | 29E | n/a | OH | Invasive | | + | 16.8 | + | 19.6 | None | |
| M10949 | 29E | n/a | PA | Invasive | | + | 14.9 | + | 19.7 | None | |
| M11139 | 29E | n/a | OH | Invasive | | + | 18.7 | + | 20.2 | None | |
| M11259 | 29E | n/a | CA | Invasive | | + | 14.1 | + | 17.3 | None | |
| M11260 | 29E | n/a | CA | Invasive | | + | 14.2 | + | 16.7 | None | |
|  |  |  |  |  | |  |  |  |  |  | |
|  |  |  |  |  | |  |  |  |  |  | |
| ^1^ Serogroup-specific PCR testing was performed to detect serogroups A, B, C, W135, X, and Y; isolates not positive for  one of these serogroups were termed NG by PCR. Serogroup-specific PCR was not performed on carriage study  isolates that were *ctrA*-negative. | | | | | | | | | | | |
| ^2^All *ctrA* C_t_ values were generated at CDC using a Stratagene Mx3005P machine except the MD carriage isolates,  which were tested at the MD Department of Health and Mental Hygiene using either an Applied Biosystems 7500 or  7700 machine. | | | | | | | | | | | |
| ^3^ All *sodC* results were an average of 2 C_t_ values except M17304, which is an average of 4 C_t_s.  ^4^ nd, not done. | | | | | | | | | | |  |
| ^5^ MLEE results for the isolates collected in the Kellerman et al. carriage study were previously reported (21). | | | | | | | | | | | |
| ^6^ MLST, Where ST is given, this isolate was previously characterized (12); where no ST given,  MLST was performed at CDC and was consistent with identification as Nm. | | | | | | | | | | | |
| ^7^ LH, MLST consistent with identification as Nm (Lee Harrison or Susanna Schmink, personal communication). | | | | | | | | | |  | |
| ^8^ There is no 29E PCR serogrouping assay currently in use in the CDC Meningitis Laboratory. However, this isolate was  negative by real-time PCR for serogroup A, B, C, W135, X, and Y genes. | | | | | | | | | | | |
| ^9^ Invasive isolates included those from CSF, blood, joint fluid, or autopsy tissue. | | | | | | | | | | | |
| ^10^ Non-invasive isolates included carriage isolates that were not from the Kellerman et al. or Clark et al. carriage studies  and also included those from sputum or oral swab specimens that were sent to CDC.  ^11^ ABCs, Collected by the Active Bacterial Core Surveillance (ABCs) Team of the indicated state through  the Emerging Infections Program Network, CDC. | | | | | | | | | | | |
| ^12^ There is no Z PCR serogrouping assay currently in use in the CDC Meningitis Laboratory. However, this isolate  was negative by real-time PCR for serogroup A, B, C, W135, X, and Y genes. | | | | | | | | | | | |
| ^13^ M0330 is a reference strain isolated in 1966. | | | | |  |  |  |  |  |  | |
