# Supplementary material for: sodC-Based Real-Time PCR for Detection of Neisseria meningitidis
Source: PLoS One. 2011 May 5;6(5):e19361. doi: 10.1371/journal.pone.0019361 (PMC3088665; doi:10.1371/journal.pone.0019361)
Supplement: Table S3 — Nm sodC sequences generated in this study. (DOCX) [file pone.0019361.s004.docx]

Table S3. Nm *sodC* sequences generated in this study.
