# Supplementary material for: sodC-Based Real-Time PCR for Detection of Neisseria meningitidis
Source: PLoS One. 2011 May 5;6(5):e19361. doi: 10.1371/journal.pone.0019361 (PMC3088665; doi:10.1371/journal.pone.0019361)
Supplement: Table S4 — Two-by-two contingency tables that were the basis for the calculation of sodC PCR sensitivity and specificity compared to culture and ctrA PCR. (a) Comparison of sodC PCR to culture for 140 clinical specimens. (b) Comparison of sodC PCR to ctrA PCR for 157 clinical specimens. (DOCX) [file pone.0019361.s005.docx]

|  |  | *sodC* PCR | |
| --- | --- | --- | --- |
|  |  | + | - |
| culture | + | 1 | 0 |
|  | - | 16 | 123 |

Table S4. Two-by-two contingency tables that were the basis for the calculation of *sodC* PCR sensitivity and specificity compared to culture and *ctrA* PCR.

1. Comparison of *sodC* PCR to culture for 140 clinical specimens.
2. Comparison of *sodC* PCR to *ctrA* PCR for 157 clinical specimens.

|  |  | *sodC* PCR | |
| --- | --- | --- | --- |
|  |  | + | - |
| *ctrA* PCR | + | 21 | 0 |
|  | - | 4 | 132 |
